# Spatial heterogeneity reveals an evolutionary signature predicting therapeutic response and clinical outcomes in hepatocellular carcinoma

**DOI:** 10.3389/fbinf.2025.1669236

**Published:** 2025-08-18

**Authors:** Shangyi Luo, Li Liu, Yang Sun, Jian Shi, Yajing Zhang

**Affiliations:** ^1^ Interdisciplinary Institute for Medical Engineering, Fuzhou University, Fuzhou, Fujian, China; ^2^ Clinical Oncology School of Fujian Medical University, Fujian Cancer Hospital, Fuzhou, Fujian, China; ^3^ School of Basic Medical Sciences, Chongqing Medical University, Chongqing, China; ^4^ NHC and CAMS Key Laboratory of Molecular Probe and Targeted Theranostics, Harbin Medical University, Harbin, Heilongjiang, China; ^5^ Heilongjiang Province Key Laboratory of Child Development and Genetic Research, Harbin Medical University, Harbin, Heilongjiang, China

**Keywords:** hepatocellular carcinoma, multi-region sequencing, expression dynamics, tumor evolution, prognostication

## Abstract

**Introduction:**

Intra-tumoral heterogeneity is a prominent characteristic of hepatocellular carcinoma (HCC). However, it remains unexplored whether intra-tumoral transcriptomic differences can capture crucial information regarding HCC evolution and be utilized to derive a predictive signature for patient’s clinical trajectories.

**Methods:**

We quantified transcriptomic heterogeneity using four multiregional HCC cohorts comprising 172 samples from 37 patients, and validated transcriptomic heterogeneity and spatial dynamics using multiregional single-cell transcriptomic profiling of 110,817 cells from 34 liver specimens. The HCC evolutionary signature (HCCEvoSig) was developed and assessed across six cross-platform HCC cohorts.

**Results:**

Genes exhibiting high intra- and inter-tumor expression variation were significantly enriched in a gene set associated with HCC prognosis, from which we developed and validated a reproducible and robust transcriptomic signature, HCCEvoSig. Multiregional single-cell data confirmed the high intra- and inter-tumoral heterogeneity of HCCEvoSig genes across different cell types, and importantly, demonstrated that the dysregulation of HCCEvoSig genes exhibited a geospatially gradual transition from the non-tumor region to the tumor border and tumor core, as well as from non-malignant to malignant epithelial cells. HCCEvoSig showed significant positive associations with adverse features of HCC, and a high HCCEvoSig risk score predicted increased risks of disease progression and mortality, independent of established clinicopathological indices. Furthermore, HCCEvoSig outperformed 15 published signatures in discriminative ability and prognostic accuracy, particularly regarding 1-year survival rates. Notably, HCCEvoSig demonstrated predictive utility for responses to immunotherapy and trans-arterial chemoembolization. Additionally, we established a well-calibrated predictive nomogram that integrates HCCEvoSig and TNM stage to generate an individualized numerical probability of mortality.

**Conclusion:**

Our study reveals that regional transcriptional heterogeneity within tumors is substantial enough to capture survival signals, and the constructed and validated HCCEvoSig provides reliable prognostic information for HCC patients.

## Introduction

Primary liver cancer is the sixth most commonly diagnosed cancer and the third leading cause of cancer-related death worldwide (Sung et al., 2021). Hepatocellular carcinoma (HCC) is the most dominant histological form of liver cancer, accounting for 75%–85% of cases (Vogel et al., 2022). Over the past 2 decades, significant progress has been made in the treatment of HCC, driven by the development of new molecular targeted therapies, immune checkpoint inhibitors (ICIs), and trans-arterial chemoembolization (TACE) (Singal et al., 2023; Brown et al., 2023). Despite these advancements, the prognosis for patients with HCC remains poor and varies significantly among individuals, with a relative 5-year survival rate of approximately 18% (Vogel et al., 2022), which may be attributed to tumor evolution induced by treatment (Craig et al., 2020). However, if detected at an early stage, surgical resection offers a favorable prognosis, with 5-year survival rates exceeding 70%. Accurate stratification reflecting the prognosis and treatment response of HCC patients is crucial for disease surveillance and the selection of treatment strategies. Therefore, there is an urgent need to develop reliable biomarkers and models that can accurately predict HCC prognosis and identify patients most likely to benefit from drug-based therapies. Considerable effort has been devoted to establishing such stratification models using patient’s clinical and pathological characteristics (Duseja, 2014). Currently, several classification systems, including the American Joint Committee on Cancer (AJCC) TNM system, the Cancer of the Liver Italian Program (CLIP), and the Barcelona Clinic Liver Cancer (BCLC) staging, have been developed and implemented in clinical practice. While these assessment approaches have proven useful, they exhibit various limitations in patient stratification and provide limited predictive accuracy (Ganesan and Kulik, 2023). Furthermore, they do not account for the biological characteristics of HCC that contribute to clinical heterogeneity, highlighting the need for improvement.

Advances in genome-wide expression profiling technology have significantly enhanced our understanding of HCC biology. Many studies assessed the prognostic abilities of gene signatures, and identified numerous predictive signatures that were nominally able to predict prognosis (Kim et al., 2012; Villa et al., 2016; Sun et al., 2024; Luo et al., 2023); however, none have yet entered clinical practice. These studies predominantly focused on the interrogation of single tissue samples from individual tumors, without concerning the evolutionary nature of tumor, thus limiting the ability to infer disease pathogenesis, and to correlate molecular findings with the clinical trajectory of individual patients. Recent studies have revealed that the clinical trajectory of HCC patients is largely determined by the most aggressive fraction of tumor cells, a phenomenon termed the ‘bad apple’ effect, wherein a bad apple spoils the whole barrel (Chen et al., 2024; Zhai et al., 2022). Additionally, Losci et al. revealed that intra-tumoral transcriptomic differences could capture the evolutionary information of tumors (Losic et al., 2020). These clues highlight the importance of obtaining data from multiple regions of a tumor, suggesting that multiregional transcriptomic analysis may aid in identifying significant transcriptomic signatures that can forecast patient’s clinical trajectories.

In the current study, we examined 172 multiregional transcriptomic profiles from 37 HCC patients to analyze intra- and inter-tumoral expression dynamics, and found genes exhibiting both high intra- and inter-tumoral expression variation were significantly enriched in prognostic information for HCC. Subsequently, we devised a *de novo* strategy to develop a transcriptomic signature, termed the HCC evolutionary signature (HCCEvoSig), which captured critical information regarding tumor evolution and provided more reliable risk estimates for HCC patients. The dynamic transcriptomic changes of HCCEvoSig genes were further validated through multiregional single-cell transcriptomic profiling of 110,817 cells from 34 liver specimens. We assessed and validated the prognostic and predictive accuracy of this classifier in five independent cohorts, involving a total of 765 HCC patients. Notably, our results demonstrated the predictive utility of HCCEvoSig for responses to immunotherapy and TACE in two retrospective HCC cohorts. Additionally, we compared its prognostic and predictive efficacy with 15 previously reported HCC prognostic models, and established a well-calibrated nomogram based on this classifier and the TNM staging system, providing a more individualized approach to predict prognostic information for HCC patients.

## Materials and methods

### Multiregional gene expression data of HCC

Following data preprocessing, multiregional gene expression data of HCC from four studies were compiled, encompassing a total of 172 samples from 37 patients (Supplementary Table S1). The MultiRRnaSeq1 cohort consisted of RNA-seq data from seven patients, comprising 33 tumoral regions (mean of 4.7 tumor regions per patient, range: 3–5), derived from the study by Losic et al. (2020). The MultiRRnaSeq2 cohort included data from 14 patients, with a total of 75 tumoral regions (mean of 5.4 tumor regions per patient, range: 3–10), obtained from the study by Yang et al. (2022). The MultiRRnaSeq3 cohort comprised data from 11 patients, with 39 tumoral regions (mean of 3.5 tumor regions per patient, range: 3–5), originating from the study by Shen et al. (2020). In addition, Agilent mRNA transcriptome profiles were curated from another study (Shi et al., 2017), consisting of 25 tumor samples from five patients with HCC (designated as the MultiRArray cohort; five regions per patient). Detailed information on data collection, filtering, and normalization is available in the Supplementary Materials.

### Acquisition of HCC cohorts with survival information

Six HCC cohorts with clinical follow-up information were compiled, encompassing a total of 1,120 HCC specimens and 693 normal specimens from 1,149 patients (Supplementary Table S2). These included four sequencing-based cohorts (TCGA-LIHC (Cancer Genome Atlas Research Network, 2017), ICGC-LIRI-JP (Fujimoto et al., 2016), CHCC-HBV (Gao et al., 2019), and Mongolia-HCC (Candia et al., 2020)) and two microarray-based cohorts (FULCI-HCC (Roessler et al., 2010) and NCI-HCC (Lee et al., 2006)). Detailed information on data collection, filtering, and normalization is available in the Supplementary Materials.

### Compilation of HCC cohorts undergoing trans-arterial chemoembolization

Microarray-based cohorts of HCC patients treated with TACE were obtained from the GEO database under accession numbers GSE104580 (Singapore-HCC-TACE) and GSE14520 (FULCI-HCC-TACE). The Singapore-HCC-TACE cohort originated from a continuing study, and included 147 patients with unresectable HCC and preserved baseline liver function, comprising 81 tumor tissues from TACE responders and 66 from non-responders. All samples were obtained from tumor biopsies collected prior to TACE treatment. Patients who achieved a complete or partial response were classified as responders, while those with stable or progressive disease were considered non-responders (Shi et al., 2013). The FULCI-HCC-TACE cohort was prospectively recruited at the Liver Cancer Institute of Fudan University and included 247 patients who underwent curative-intent resection between 2002 and 2003. Patients lacking relevant clinical data or those receiving non-TACE adjuvant or recurrence therapies were excluded (Fako et al., 2019). For downstream analysis, only data generated using the GPL571 platform were included, consisting of 71 patients who received adjuvant TACE after liver resection and 28 patients who received TACE following tumor recurrence.

### Collection of previously published HCC prognostic signatures

Fifteen previously published HCC prognostic gene expression signatures (ProGESigs), each associated with a specific formula, were collected. Detailed information for each signature and the strategy for its application are presented in Supplementary Table S4 and in the Supplementary Methods of the Supplementary Materials.

### Establishment and validation of the HCC prognostic signature

A five-step analysis pipeline was developed to construct and validate an HCC evolution-related prognostic model based on six expression datasets. First, gene heterogeneity scores were calculated using transcriptome data from four multi-region sequencing studies of HCC (see Supplementary Methods for details), and genes exhibiting high inter- and intra-tumor heterogeneity were selected and integrated. Second, dysregulated genes were identified using 175 paired tumor-normal samples from the ICGC-LIRI-JP cohort. Differential expression analysis was performed using the R package DESeq2, with genes exhibiting an absolute log 2-fold change >1.0 and adjusted *p* value <0.05 considered differentially expressed. Third, univariate Cox regression analysis was conducted to identify genes significantly associated with overall survival (OS) in the TCGA-LIHC cohort. Statistically significant was defined as a *p*-value <0.05. Fourth, the elastic-net algorithm was employed to refine the candidate genes selected by the aforementioned criteria, through removing redundancies and selecting the most informative prognostic markers for HCC. Specifically, the TCGA-LIHC training dataset was subsampled 1,000 times, and genes repeatedly selected by least absolute shrinkage and selection operator (LASSO) penalized Cox regression against OS were identified using the R package glmnet. Finally, an HCC evolutionary signature was constructed through multivariate Cox regression analysis. The risk score for each patient was calculated as a linear combination of gene expression values, weighted by the model coefficients fitted in the training cohort. Patients were then dichotomized into high- and low-risk groups based on the median risk scores in each cohort. The predictive performance of the signature was assessed via Harrell’s concordance index (C-index) and time-dependent receiver operating characteristic (ROC) curve analysis.

### Analysis of multiregional scRNA-seq data of liver cancer

Multiregional single-cell transcriptomic data from seven liver cancer patients were analyzed, including four patients with primary HCC and three with primary intrahepatic cholangiocarcinoma (iCCA) (Ma et al., 2022). These data were obtained from the GEO database under accession number GSE189903. Specifically, three not-adjacent samples from the tumor core (T1, T2, and T3), one sample from the tumor border (B), and one sample from the adjacent non-tumor tissue (N), which was in close proximity to the tumor, were included. The Seurat package in R was utilized to conduct quality control (QC) and downstream analyses. Details of the QC process, including quality checks, data filtering, and the identification and removal of cellular debris, doublets, and multiplets, are provided in the Supplementary Methods.

Following quality filtering, a total of 110,817 cells were retained for downstream bioinformatic analyses using Seurat’s standard pipeline (Satija et al., 2015). First, normalization and scaling of the feature expression measurements for each cell were performed using the NormalizeData and ScaleData functions. Cell clustering was then conducted using a shared nearest neighbor graph-based method, followed by the original Louvain algorithm for modularity optimization after data dimensionality reduction through principal component analysis. Once the cell clusters were determined, their marker genes were identified using the FindMarkers function. For cluster annotation, the top marker genes were manually curated to align with canonical cell types and their associated marker genes based on literature research. Detailed information on dimensionality reduction, clustering, and cell type determination is provided in the Supplementary methods. Malignant and non-malignant epithelial cells were inferred based on large-scale chromosomal copy-number variations derived from single-cell transcriptome profiles, as described in previously published single-cell studies (Ma et al., 2022; Ma et al., 2021; Ma et al., 2019). Differential gene expression between conditions was calculated using the Wilcoxon rank-sum test with Bonferroni correction, implemented in the FindMarkers function with the following parameters: min.pct = 0.10 and logfc.threshold = 0.25. For the analysis, a minimum of 10 cells per group was required under specific conditions. Genes with logFC >0.25 and FDR <0.05 were considered differentially expressed.

### Immune infiltration and immunophenoscore analysis

The relative proportions of 22 infiltrating immune cell types were estimated using the TCGA-LIHC cohort based on CIBERSORT algorithm (Chen et al., 2018), and these cells were aggregated into broader immune cell categories, including CD8^+^ T cells, CD4^+^ T cells, B cells, NK cells, plasma cells, monocytes, macrophages, dendritic cells, mast cells and neutrophils, following the guidelines outlined by Thorsson et al. (Thorsson et al., 2018). The immunophenoscore (IPS), which predicts the likelihood of response to anti-CTLA-4 and anti-PD1 therapies by quantifying tumor immunogenicity, immunomodulators, effector cells, and suppressor cells (Charoentong et al., 2017), was obtained for HCC patients from The Cancer Immunome Atlas (TCIA, https://tcia.at/home). Additionally, the Tumor Immune Dysfunction and Exclusion (TIDE) algorithm was employed to evaluate potential responses to immunotherapy (Fu et al., 2020). TIDE scores and T cell exclusion scores for HCC patients were calculated using the TIDE Python package (https://github.com/jingxinfu/TIDEpy).

### Statistical analysis

Categorical variables were analyzed using the Chi-squared test or Fisher’s exact test, as appropriate. Pearson’s and Spearman’s correlation tests were used to assess correlations between variables, as appropriate. Survival analysis was performed using the Kaplan-Meier method, with *p*-values determined using the log-rank test. Hazard ratios, along with univariate and multivariate analyses adjusting for age, gender, AJCC stage, cirrhosis, alpha foetoprotein (AFP), and histological grade (if available), were calculated using a Cox proportional hazards model. The nomogram and corresponding calibration maps were constructed using the R package rms. Calibration plots, generated via a bootstrap method with 1,000 resamples, were used to evaluate the concordance between actual and predicted survival. The C-index was calculated using the R package Hmisc. Time-dependent ROC analysis and AUC values were calculated using the R package timeROC. All statistical analyses were two-tailed, performed using R statistical software, and statistical significance was defined as a *p*-value <0.05.

## Results

### EvoGenes represent a concentrated reservoir of prognostic information

**FIGURE 1:**
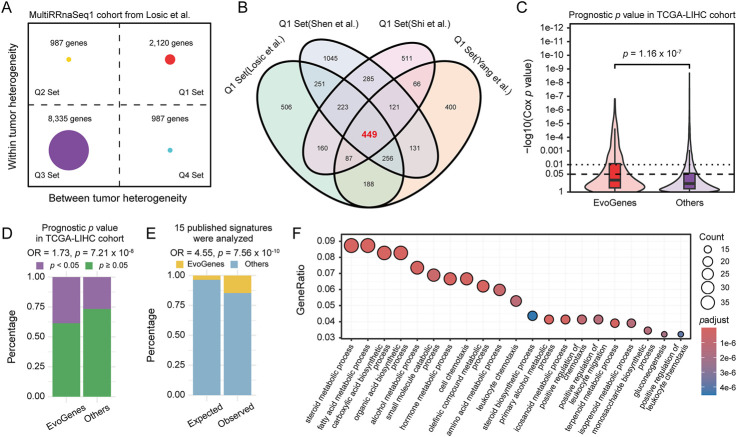
Gene Expression Heterogeneity and Prognostic Significance. **(A)** Gene expression inter- and intra-tumoral heterogeneity quadrants calculated using the MultiRRnaSeq1 cohort. The plot is divided into four quadrants (Q1, Q2, Q3, and Q4) based on the 75th percentile of inter- and intra-tumoral heterogeneity scores. **(B)** Venn diagram illustrating the consensus set of 449 genes, designated as EvoGenes, derived from four independent multiregional HCC cohorts. **(C)** Prognostic *p* values of genes in the TCGA-LIHC cohort assessed using a univariate Cox proportional hazards model, stratified by EvoGenes and other genes. Box plots represent median values, as well as the 25th and 75th percentiles, with vertical bars spanning the 5th to 95th percentiles. Statistical significance was tested using a two-sided Wilcoxon signed-rank test. **(D)** Percentage of genes with prognostic significance (univariate Cox *p* < 0.05) in the TCGA-LIHC cohort, stratified by EvoGenes and other genes in the cohort. **(E)** Stacked bar plot showing the percentage of EvoGenes in expected (all expressed genes in the MultiRRnaSeq1 cohort) versus observed (genes merged from 15 published HCC prognostic signatures) categories. Statistical significance was tested using a two-sided Fisher’s exact test. **(F)** Bubble plot displaying the Gene Ontology (GO) enrichment results for EvoGenes. The size and color of the nodes represent the number of genes and the adjusted *p*-value of each GO term, respectively.

Given the extensive transcriptomic intra-tumor and inter-tumor heterogeneity observed in previous multi-region sequencing studies of HCC (Losic et al., 2020; Yang et al., 2022; Shen et al., 2020; Shi et al., 2017), we hypothesized that intra-tumoral expression variation would capture critical information regarding tumor evolution, while inter-tumoral expression variation would reflect differences between patients. If this hypothesis holds true, genes exhibiting high levels of both types of variation would demonstrate significant prognostic value in HCC cohorts based on single biopsy samples. To explore this hypothesis, we derived inter- and intra-tumoral heterogeneity metrics for each gene using multi-region HCC samples, and stratified both heterogeneity metrics into a high and low group based on their 75th percentile, resulting in four gene heterogeneity quadrants. The analysis utilized four independent cohorts, involving transcriptomic data from a total of 172 multi-region primary HCC samples, representing 37 patients (Supplementary Table S1). Genes classified in Q1 met the desired criteria, demonstrating both high intra-tumoral variability that reflected HCC clonal evolution, and significant inter-tumoral variability that potentially served as valuable biomarkers for patient stratification. Specifically, we identified 2,120 Q1 genes from the MultiRRnaSeq1 cohort, 1,698 from the MultiRRnaSeq2 cohort, 2,761 from the MultiRRnaSeq3 cohort, and 1,902 from the MultiRArray cohort (Figure 1A; Supplementary Figure S1A-C). To minimize inter-dataset variability and enhance the accuracy of identifying genes with high intra- and inter-tumoral expression variation, we integrated Q1 genes from the four datasets, resulting in a consensus set of 449 genes (Figure 1B), designated as evolution-related genes (EvoGenes).

Prognostic significance analysis using the TCGA-LIHC RNA-seq dataset revealed that EvoGenes exhibited significantly lower *p*-values (*p* = 1.16 × 10^−7^; Figure 1C) and a higher proportion of prognosis-associated genes (Cox *p* < 0.05) compared to other genes (OR = 1.73, *p* = 7.21 × 10^−8^; Figure 1D). This prognostic superiority was consistently replicated across five independent validation cohorts, comprising three RNA-seq-based and two microarray-based gene expression datasets (total: n = 765 patients; Supplementary Table S2; Supplementary Figure S1D-M). Notably, while EvoGenes represented only 4% of all expressed genes in the MultiRRnaSeq1 cohort (449/12,429), they accounted for 15% of the genes identified in fifteen published prognostic signatures (28/192; Supplementary Table S3), demonstrating an approximately fivefold enrichment (OR = 4.55, *p* = 7.56 × 10^−10^; Figure 1E). This enrichment suggested that prior studies tended to select EvoGenes even without explicit consideration of tumor evolution. Similar results were observed in three other multiple-region cohorts (Supplementary Figure S1N). These findings provided evidence that EvoGenes were highly enriched for genes with a reproducible association with survival compared to other genes. Gene ontology enrichment analysis indicated that EvoGenes were significantly overrepresented in critical biological processes, including metabolic, immune and metastasis categories (Figure 1F; Supplementary Table S4), with similar result observed in KEGG enrichment analysis (Supplementary Figure S1O; Supplementary Table S5). Collectively, these findings suggested that EvoGenes represented a concentrated reservoir of prognostic information, providing valuable insights into tumor progression and patient outcomes.

### A de novo strategy to develop a prognostic signature linked to HCC evolution

**FIGURE 2:**
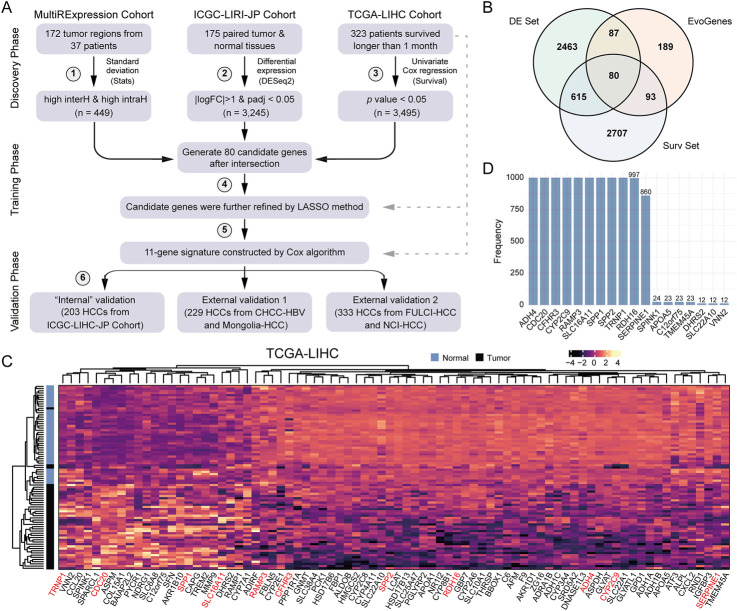
A Six-step *De Novo* Strategy to Develop HCCEvoSig. **(A)** Schematic representation summarizing the strategy to construct and validate the HCC prognostic signature, termed HCCEvoSig. **(B)** Venn diagram illustrating candidate prognostic genes with evolutionary features. EvoGenes, inherited from Figure 1B, represents genes with high inter-tumoral and intra-tumoral heterogeneity. The DE set consists of differential expression genes identified using paired tumor and normal samples from the ICGC-LIRI-JP cohort. The Surv set includes genes associated with overall survival in the TCGA-LIHC cohort. **(C)** Heatmap displaying the expression of 80 candidate genes in paired tumor and normal samples from the TCGA-LIHC cohort. Genes marked in red are components of HCCEvoSig. **(D)** Bar plot showing the frequency of gene occurrence in the model based on 1,000 random experiments.

To assess the significance of our findings for biomarker design, we devised a *de novo* six-step strategy to construct and validate a prognostic signature for HCC (Figure 2A). In the discovery phase, we utilized six expression datasets, including five RNA-seq-based and one microarray-based, encompassing 845 liver tissue samples from 535 HCC patients: (Sung et al., 2021): the aforementioned four multiple-region expression datasets, from which candidate RNA molecules exhibiting high inter- and intra-tumoral heterogeneity (i.e., EvoGenes) were derived; (Vogel et al., 2022); the ICGC-LIRI-JP HCC dataset (350 samples from 175 paired tumor-normal tissues), used to identify differentially expressed genes; and (Singal et al., 2023) the TCGA-LIHC dataset (323 HCC patients with prognostic information), from which genes associated with survival were identified. In the validation phase, we employed transcriptome data from a total of 765 HCC patients across five cohorts (Supplementary Table S2), including three RNA-seq-based cohorts (ICGC-LIRI-JP, CHCC-HBV, and Mongolian-HCC cohorts; n = 203, 159 and 70, respectively) and two microarray-based cohorts (FULCI-HCC and NCI-HCC cohorts; n = 221 and 112, respectively).

To identify genes with reproducible survival associations, we further integrated EvoGenes with differentially expressed genes (DE genes) and survival-associated genes (Surv genes) from two independent datasets (ICGC-LIRI-JP and TCGA-LIHC, respectively), resulting in a candidate gene set containing 80 genes (Figure 2B). Differential expression analysis conducted across five cohorts containing paired HCC and normal liver tissues revealed that all these 80 candidate genes were significantly differentially expressed in all cohorts (a total of 660 paired tumor-normal tissues), encompassing four RNA-seq-based datasets and one microarray-based dataset (Figure 2C, Supplementary Figure S2), validating that these genes were generally dysregulated in HCC regardless of the profiling platform, thus they might play critical roles in HCC evolution. Subsequently, we utilized the elastic-net algorithm, to refine the 80 candidate genes by removing redundancies and selecting the most informative prognostic markers for HCC. In this analysis, we subsampled the dataset 1,000 times and selected the genes that were repeatedly chosen in more than 850 iterations (Figure 2D), resulting in an 11-gene prognostic signature. Finally, an HCC evolutionary signature was derived through multivariate Cox regression analysis, which we designated as HCCEvoSig (the components of HCCEvoSig are detailed in Supplementary Table S6).

### HCCEvoSig gene expression exhibits spatial dynamics

**FIGURE 3:**
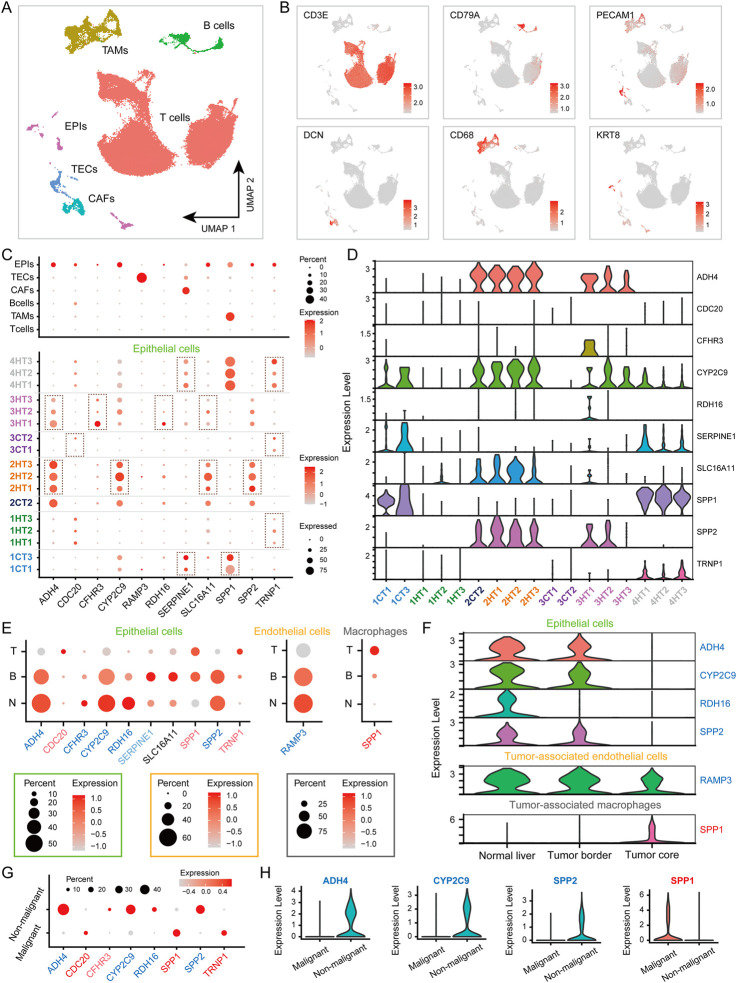
HCCEvoSig Genes Exhibit Spatial Dynamic Expression Changes. **(A)** UMAP plot of 110,817 filtered single cells, colored by their assigned cell types: T cells, tumor-associated macrophages (TAMs), B cells, epithelial cells (EPIs), cancer-associated fibroblasts (CAFs), and tumor-associated endothelial cells (TECs). **(B)** UMAP plot showing the expression of marker genes for the 6 cell types. **(C)** Dot plots illustrating the percentage of each cell type (upper) and EPIs across different patients and tumor cores (bottom) expressing HCCEvoSig genes (indicated by the size of the circle) and their scaled expression levels (indicated by the color of the circle). **(D)** Violin plot displaying the expression levels of HCCEvoSig genes in EPIs across different patients and tumor cores. **(E)** Dot plots depicting the expression of HCCEvoSig genes that are differentially expressed across geospatial regions in EPIs (left), TECs (middle) and TAMs (right). The size of the circle represents the percentage of cells expressing the gene in that specific region, while the color indicates the average expression of the gene. Genes marked in blue and red indicate downregulation and upregulation in the tumor core compared to normal tissue, consistent with bulk analysis. Light blue and light red genes did not reach the strict statistical significance threshold defined in the Methods. See Supplementary Table S9 for details. Non-tumor tissue (N); tumor border **(B)**; tumor core (T). **(F)** Violin plots for representative genes expressed in EPIs (upper), TECs (middle) and TAMs (bottom), which exhibit differential expression across geospatial regions. The color coding of the genes is consistent with that in panel **(E) (G)** Dot plots depicting the expression of HCCEvoSig genes that are differentially expressed between malignant EPIs and non-malignant EPIs. Genes marked in blue and red indicate downregulation and upregulation in malignant EPIs compared to non-malignant EPIs. Light blue and light red genes did not reach the strict statistical significance threshold defined in the Methods. See Supplementary Table S9 for details. **(H)** Violin plots of representative HCCEvoSig genes that are differentially expressed between malignant EPIs and non-malignant EPIs. The color coding of the genes is consistent with that in panel **(G)**

To explore the spatial expression patterns of HCCEvoSig genes, we analyzed multiregional single-cell transcriptomic data of 34 liver specimens from four HCC and three iCCA patients. Graph-based clustering and canonical cell marker annotation revealed six major cell types (Figures 3A,B and Supplementary Figures S3A,B): T cells (n = 87,476), tumor-associated macrophages (TAMs, n = 7,380), B cells (n = 3,094), epithelial cells (EPIs; n = 2,250), cancer-associated fibroblasts (CAFs, n = 1,530), and tumor-associated endothelial cells (TECs, n = 1,332). The cell identities identified here were highly consistent with those reported in the original study (Ma et al., 2022) (Supplementary Figure S3C). A dot plot displaying the expression of HCCEvoSig genes indicated that ten of eleven HCCEvoSig genes were expressed in EPIs with relatively low expression levels. *RAMP3*, *SERPINE1* and *SPP1* were primarily expressed in TECs, CAFs and TAMs, respectively. Notably, we did not observe expression of HCCEvoSig genes in B cells and T cells. The relatively low expression levels of HCCEvoSig genes in specific cell types implied that these genes might harbor high transcriptomic heterogeneity both intra-tumorally and inter-tumorally. When examining the expression of HCCEvoSig genes in different tumor cores (T1, T2, and T3) across various patients, we indeed found that those HCCEvoSig genes expressed in EPIs exhibited marked variability in intra-tumor and inter-tumor contexts (Figures 3C,D). Similar phenomena were observed for *RAMP3*, *SERPINE1* and *SPP1* (Supplementary Figures S3D,E) in TECs, CAFs and TAMs, respectively. We also analyzed HCC patients separately to avoid potential tumor type bias, and found consistent results (Supplementary Figures S3F-G).

Given that HCCEvoSig was constructed with considering the differential expression between tumor and normal samples, we further explored the spatial dynamics of HCCEvoSig gene expression from adjacent non-tumor tissue (N) to tumor border (B) and tumor core (T). We found that HCCEvoSig genes exhibited different expression levels between normal and tumor tissues, largely consistent with bulk analysis (Figure 3E; Supplementary Table S7). Interestingly, we observed continuous dynamic changes in expression from normal tissue to tumor core (Figures 3E,F). Specifically, from N to B and T, *ADH4*, *CFHR3*, *CYP2C9*, *RDH16* and *SPP2* showed sustained downregulation in EPIs, while *CDC20* and *TRNP1* exhibited sustained upregulation. The expression of *RAMP3* in TECs demonstrated continuous downregulation, whereas *SPP1*, expressed in both TAMs and EPIs, showed sustained upregulation. An exception was *SLC16A11*, which demonstrated increased expression in tumor samples in bulk analysis but did not show significant upregulation in the EPIs of tumor core; however, we noted its increased expression in B compared to N (Figure 3E). Another exception was *SERPINE1*, which showed decreased expression in bulk analysis. We found that the expression of *SERPINE1* in CAFs slightly increased from N to T (Supplementary Figure S4A), while in EPIs, *SERPINE1* exhibited decreased expression in T compared to N (Figure 3E). When analyzing data from HCC patients alone, we obtained similar results (Supplementary Figure S4B,C; Supplementary Table S8). Furthermore, we explored HCCEvoSig gene expression in malignant EPIs, and found that genes exhibiting sustained upregulation or downregulation in EPIs were further upregulated or downregulated in malignant EPIs compared to non-malignant EPIs (Figures 3G,H; Supplementary Table S9). Consistent results were observed when analyzing data from HCC patients alone (Supplementary Figure S4D-E; Supplementary Table S10). Collectively, HCCEvoSig genes demonstrated dynamic transcriptomic changes in intra-tumoral and inter-tumoral contexts, and importantly, exhibited geospatially consistent evolution from N to B, T, suggesting that they underlined the progression of HCC.

### Prognostic implications of HCCEvoSig in HCC across diverse datasets

**FIGURE 4:**
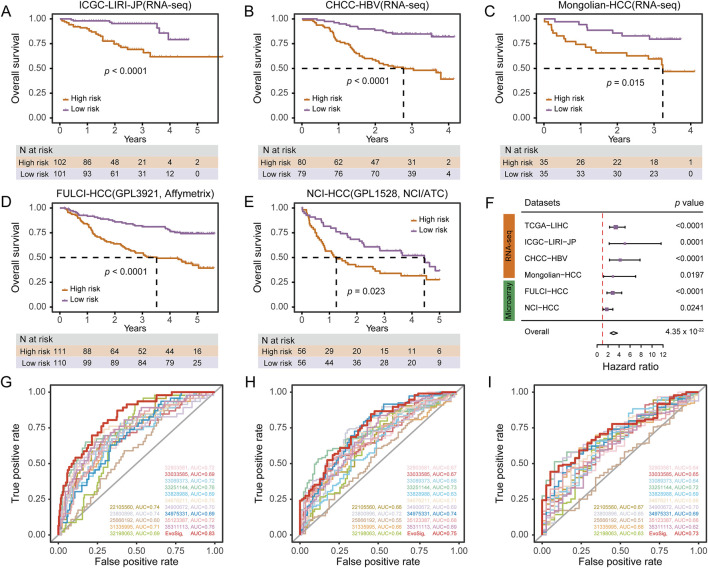
HCCEvoSig Demonstrates Robust and Reproducible Prognostic Efficacy Across HCC Cohorts and Profiling Platforms. **(A–E)** Kaplan-Meier estimations of overall survival in HCC patients, stratified by HCCEvoSig risk scores, across independent RNA-seq-based validation cohorts: ICGC-LIRI-JP **(A)**, CHCC-HBV **(B)**, and Mongolian-HCC **(C)**, as well as independent microarray-based validation cohorts: FULCI-HCC **(D)**, and NCI-HCC **(E)**. Patients in each dataset are categorized into high-risk and low-risk groups based on the median HCCEvoSig risk score. The dashed lines indicate the subgroup-specific median overall survival (OS). **(F)** Forest plot summarizing the prognostic power of HCCEvoSig in a meta-analysis across all six cohorts. Univariate Cox regression analysis was performed for each dataset; hazard ratios, 95% confidence intervals, and *p* values are presented. The diamond indicates the hazard ratio for the meta-analysis. **(G–I)** Time-dependent ROC curves comparing HCCEvoSig with 15 other established HCC signatures regarding 1-year **(G)**, 3-year **(H)**, and 5-year **(I)** survival predictions.

To investigate the prognostic performance of HCCEvoSig, we firstly dichotomized TCGA-LIHC patients using the median risk score, and found that the HCCEvoSig risk score was significantly positively associated with mortality (log-rank test: *p* < 0.0001; univariate Cox regression: *p* < 0.0001, HR = 3.40; Supplementary Figure S5A; Supplementary Table S11). The median OS interval for patients with high HCCEvoSig scores was 2.46 years (95% CI: 1.76–3.82), whereas the median OS for patients with low HCCEvoSig scores was 6.93 years (95% CI: 5.83-NA). Subsequently, we applied the HCCEvoSig weights trained in the TCGA-LIHC discovery cohort to three additional independent RNA-seq-based HCC datasets. Consistent results were observed across all datasets, indicating that patients with high HCCEvoSig risk scores had shorter median survival times (Figures 4A–C; Supplementary Tables S12-14 log-rank test: *p* < 0.0001; univariate Cox regression: *p* = 0.0001, HR = 5.06 in the ICGC-LIRI-JP cohort; log-rank test: *p* < 0.0001; univariate Cox regression: *p* < 0.0001, HR = 4.20 in the CHCC-HBV cohort; log-rank test: *p* = 0.015; univariate Cox regression: *p* = 0.0197, HR = 2.88 in the Mongolian-HCC cohort).

Moreover, we included two microarray-based HCC expression datasets to further evaluate the prognostic power of HCCEvoSig. We initially anticipated that the performance of HCCEvoSig might be diminished due to the cross-platform application of weights trained on TCGA-LIHC transcriptome sequencing data. However, HCCEvoSig remained significantly associated with survival in both microarray datasets (Figures 4D,E; log-rank test: *p* < 0.0001; univariate Cox regression: *p* < 0.0001, HR = 2.84 in the FULCI-HCC cohort; log-rank test: *p* = 0.0230; univariate Cox regression: *p* = 0.0241, HR = 1.76 in the NCI-HCC cohort). In a meta-analysis considering all training and testing cohorts (combined: n = 1,088 HCC patients), HCCEvoSig also demonstrated a significant association with patient outcomes (Figure 4F; univariate Cox regression: *p* = 4.35 × 10^−22^, HR = 2.98 [2.39–3.72]). Furthermore, we observed that HCC patients with higher HCCEvoSig risk scores exhibited significantly faster disease progression compared to those with lower scores across three datasets (Supplementary Figure S5B-D). These results demonstrated the reproducible prognostic performance and concordance of HCCEvoSig across multiple cohorts from different profiling platforms, suggesting that a prognostic signature robust to variations in cohort characteristics and expression profiling technologies could be achieved by capturing the transcriptomic phenotype of HCC evolution.

### HCCEvoSig demonstrates independent and superior prognostic performance

In four out of five training and validation datasets with available clinicopathological factors, HCCEvoSig was significantly associated with OS in multivariate Cox proportional hazards analysis, adjusting for age, sex, TNM stage, histological grade, cirrhosis, and AFP (Supplementary Table S11-15, TCGA-LIHC: *p* = 0.0008, HR = 3.11 [1.60–6.06]; ICGC-LIRI-JP: *p* = 0.0031, HR = 3.71 [1.56–8.86]; CHCC-HBV: *p* = 0.0002, HR = 3.45 [1.82–6.55]; and FULCI-HCC: *p* = 0.0038, HR = 2.05 [1.26–3.34]). The non-significant result obtained in the Mongolian-HCC cohort may be attributed to the limited sample size when multiple variables were considered (fewer than 50 samples for multivariate analysis; Supplementary Table S14). Notably, HCCEvoSig remained a significant prognostic model even when TNM stage was replaced with BCLC stage or CLIP stage, which are collinear with TNM stage (Supplementary Tables S16-18). This analysis suggested that HCCEvoSig provided independent prognostic value beyond established clinicopathological indices.

As previously reported, gene expression signatures nominally have excellent performance in predicting outcomes for HCC patients. Here, we evaluated the discrimination and prognostic accuracy of 15 established multigene HCC signatures alongside our HCCEvoSig. Time-dependent ROC curve analysis and C-index calculations indicated that HCCEvoSig was either superior to or comparable with the other 15 models (Supplementary Table S3) in terms of 1-, 3-, and 5-year survival prediction (Figures 4G–I; Supplementary Table S19). Notably, HCCEvoSig unequivocally outperformed the other models in terms of the AUC for 1-year survival (0.83, 95% CI: 0.77–0.89) and C-index (0.74, 95% CI: 0.70–0.79) (Figure 4G; Supplementary Table S19). These results demonstrated that HCCEvoSig not only maintained robust prognostic performance across diverse cohorts but also represented a significant advancement over existing multigene signatures in predicting short-term survival outcomes for HCC patients.

### HCCEvoSig correlates with aggressive clinical and Molecular Features

**FIGURE 5:**
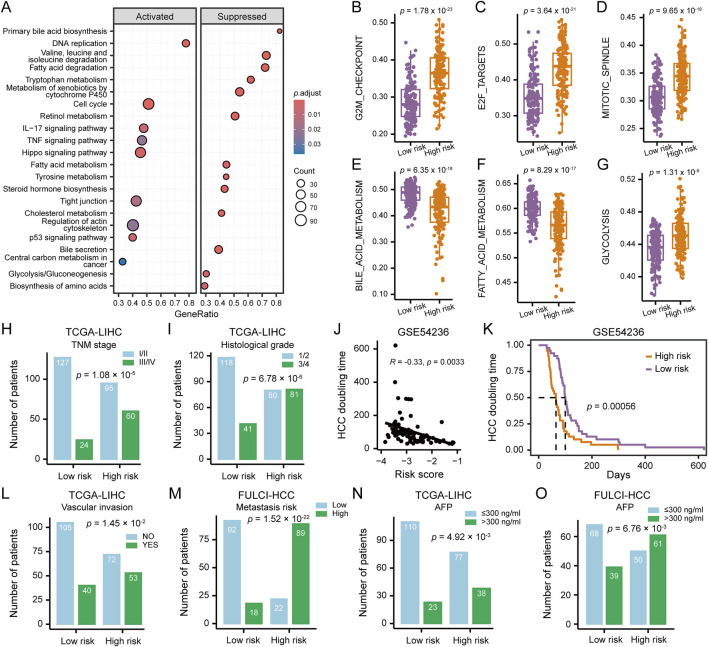
Clinicopathological and Molecular Features of HCC Patients Stratified by HCCEvoSig Risk Score. **(A)** Gene set enrichment analysis illustrating upregulated and downregulated pathways in HCC tumors with high HCCEvoSig scores, assessed in the TCGA-LIHC cohort. **(B–G)** Box plots showing differences in representative pathway activities between HCC tumors with high and low HCCEvoSig risk scores in the TCGA-LIHC cohort. The center line represents the median, and the box bounds indicate the 25th and 75th percentiles. **(H–I)** Bar charts showing the distribution of HCC patients across different TNM stages **(H)** and histological grades **(I)** within the high and low HCCEvoSig risk groups in the TCGA-LIHC cohort. **(J)** Correlation between HCCEvoSig risk scores and tumor volume doubling time calculated from imaging data in the GSE54236 cohort. Pearson’s correlation test was used for correlation analysis. **(K)** Kaplan-Meier analysis of tumor volume doubling time in HCC patients from the GSE54236 cohort, stratified by HCCEvoSig risk score. **(L–O)** Bar charts showing the distribution of HCC patients with different vascular invasion statuses (L, TCGA-LIHC), varying metastatic risks (M, FULCI-HCC), and different serum AFP levels (N, TCGA-LIHC; O, FULCI-HCC) within the high and low HCCEvoSig risk groups.

Exploring the clinicopathological and biological underpinnings of the HCCEvoSig signature using the TCGA-LIHC cohort, we found that HCC tumors with high HCCEvoSig scores exhibited elevated activity in cell cycle-related pathways (Figure 5A; Supplementary Table S20), such as DNA replication, cell cycle, and p53 signaling pathway, along with enhanced central carbon metabolism. In contrast, these tumors showed suppressed lipid metabolism and amino acid metabolism compared to those with low HCCEvoSig scores. Assessment of tumor hallmark activities further reinforced these findings, revealing significantly activated cell-cycle related pathways, including the E2F targets, G2M checkpoint, and mitotic spindle pathways, alongside enhanced glycolysis, while fatty acid metabolism was suppressed (*p* < 0.0001; Figures 5B–G; Supplementary Table S21). Additionally, significantly mutated gene analysis performed in the TCGA-LIHC cohort revealed that patients with high HCCEvoSig scores had a significantly higher probability of *TP53* mutations (50% in high vs 13% in low HCCEvoSig risk group, *p* = 1.00 × 10^−12^; Supplementary Figure S6A), which was further confirmed in three independent validation cohorts (Supplementary Figure S6B-D; ICGC-LIRI-JP, CHCC-HBV and Mongolian-HCC cohorts). Conversely, we found that *CTNNB1* mutations were more prevalent in the low HCCEvoSig risk group, although this was statistically significant only in the ICGC-LIRI-JP and CHCC-HBV cohorts (Supplementary Figure S6B, C). These results were consistent with previous studies indicating that *TP53* mutations were significantly enriched in high-risk and poorly differentiated HCC, whereas *CTNNB1* mutations were predominantly associated with low-risk and well-differentiated HCC (Calderaro et al., 2017).

Clinical factors analysis revealed that higher TNM stage (proportion of patients at stage III/IV: 39% in high vs 16% in low HCCEvoSig risk group, *p* = 1.08 × 10^−5^; Figure 5H) and histological grade (proportion of patients at grade 3/4: 50% in high vs 26% in low HCCEvoSig risk group, *p* = 6.78 × 10^−6^; Figure 5I) were significantly enriched in the high HCCEvoSig risk group. In the ICGC-LIRI-JP, CHCC-HBV and FULCI-HCC cohorts, we validated patients with advanced HCC, characterized by high histological grade, TNM stage, BCLC stage, or CLIP stage, exhibited a significant increase in HCCEvoSig risk scores (*p* < 0.005; Supplementary Figures S6E-K). Moreover, we found that HCCEvoSig risk scores were significantly negatively correlated with HCC doubling times, which were calculated based on imaging data in the GSE54236 cohort (*R* = −0.33, *p* = 0.0033; Figure 5J), implying patients with high HCCEvoSig scores had significantly shorter HCC doubling times (log-rank test: *p* = 0.0006; Figure 5K). A high HCCEvoSig risk score also predicted an increased risk of vascular invasion (*p* = 1.45 × 10^−2^; Figure 5L) and metastasis (*p* = 1.52 × 10^−22^; Figure 5M) in the TCGA-LIHC and FULCI-HCC cohorts, respectively, with consistent results obtained in the ICGC-LIRI-JP cohort (Supplementary Figures S6L-M). Additionally, the serum level of AFP was significantly positively associated with HCCEvoSig risk scores in both training and validation datasets across RNA-seq-based and microarray-based platforms (Figures 5N,O and Supplementary Figure S6N-O; *p* < 0.01 for TCGA-LIHC, CHCC-HBV, Mongolian-HCC, and FULCI-HCC cohorts). These results indicated that aggressive characteristics, including advanced tumor status, faster tumor growth rates and higher invasive and metastatic potential, were presented in HCC patients with high HCCEvoSig scores.

### Predictive practicability of HCCEvoSig for therapeutic response

**FIGURE 6:**
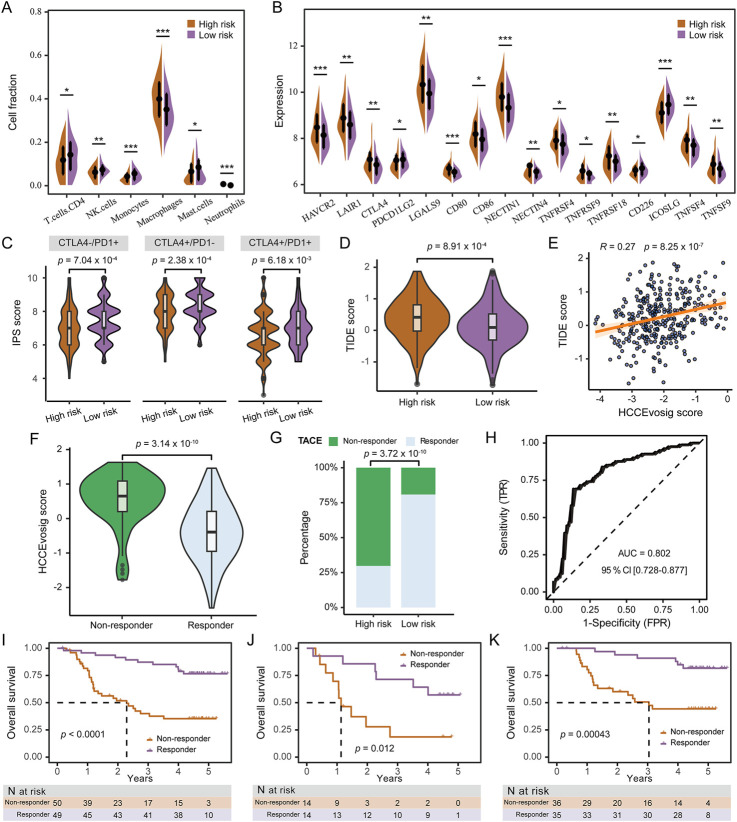
Predictive Performance of HCCEvoSig in Treatment Response. **(A)** Violin plot illustrating differences in immune cell infiltration between the high and low HCCEvoSig risk groups. Statistical significance: *p < 0.05; **p < 0.01; ***p < 0.001. **(B)** Violin plot showing the expression levels of immune checkpoint molecules across HCCEvoSig risk groups. Statistical significance: *p < 0.05; **p < 0.01; ***p < 0.001. **(C)** Comparison of immunophenoscore (IPS) scores in response to immune checkpoint blockade between the low and high HCCEvoSig risk groups. **(D)** Boxplot showing differences in TIDE scores between high and low HCCEvoSig risk groups. **(E)** Scatter plot illustrating the linear correlation between the HCCEvoSig risk score and TIDE score. **(F)** Boxplot comparing HCCEvoSig scores between TACE responders and non-responders. **(G)** Proportion of TACE responders and non-responders in the high and low HCCEvoSig risk groups. **(H)** ROC curve showing the predictive accuracy of the HCCEvoSig model for TACE response in the Singapore-HCC-TACE cohort. **(I–K)** OS comparison between predicted responders and non-responders receiving either adjuvant or post-recurrence TACE **(I)**, adjuvant TACE treatment **(J)**, and post-recurrence TACE treatment **(K)**. The dashed lines indicate the subgroup-specific median OS.

Several treatments have been proposed for advanced HCC, including immunotherapy, molecular targeted therapy and TACE. As a locoregional therapy, TACE can induce tumor cell necrosis, facilitating the release of tumor-associated antigens and pro-inflammatory cytokines, which in turn remodels the tumor microenvironment (TME), and modulates it into a state more conducive to the efficacy of ICIs in antitumor response (Pinato et al., 2021; Ren et al., 2025). We firstly assessed the correlation between the infiltration abundance of immune cells inferred by the CIBERSORT algorithm and the HCCEvoSig risk score using the TCGA-LIHC cohort. The results showed that cells with antitumor effects, such as CD4^+^ T cells, NK cells, monocytes, and plasma cells, were negatively associated with the HCCEvoSig risk score, whereas cells with pro-tumor functions, such as macrophages and neutrophils, were positively correlated (Figure 6A). In addition to immune cell infiltration, another factor influencing the efficacy of ICIs is the expression level of immune checkpoint molecules. Therefore, we evaluated the association between immune checkpoint expression and HCCEvoSig risk scores, and found that numerous markers exhibited significantly different expression levels between the high- and low-risk groups (Figure 6B). We further investigated whether the HCCEvoSig risk score could serve as a predictor of response to ICIs in patients with HCC. The IPS is a widely applied indicator for predicting immunotherapy responsiveness. In this study, we observed that IPS scores for anti-PD-1 and anti-CTLA-4 therapies were significantly higher in the low-risk group (Figure 6C). Finally, we demonstrated that high-risk patients exhibited enhanced immune evasion capacity and elevated TIDE scores (Figures 6D,E: Supplementary Figure S7A, B). These findings indicated that the HCCEvoSig model held potential predictive value for assessing the efficacy of ICI therapy.

To further evaluated the clinical utility of the HCCEvoSig model, we analyzed two retrospective cohorts of HCC patients who underwent TACE: the FULCI-HCC-TACE and Singapore-HCC-TACE cohorts. In the Singapore-HCC-TACE cohort, TACE non-responders exhibited significantly higher HCCEvoSig risk scores (Figure 6F), and were predominantly enriched in the high-risk group defined by HCCEvoSig (Figure 6G). The HCCEvoSig model demonstrated superior performance in distinguishing responders from non-responders, with an AUC of 0.80 (95% CI: 0.73–0.88; Figure 6H). In the FULCI-HCC-TACE cohort, patients were stratified into responder and non-responder groups based on the HCCEvoSig model using the median risk score as the threshold. As shown in Figure 6I, the predicted responder group exhibited significantly longer OS compared to the non-responder group (log-rank test: *p* < 0.0001). Among patients who received adjuvant TACE treatment, the model successfully identified a group with markedly prolonged OS (log-rank test: *p* = 0.0004; Figure 6J). Likewise, among patients receiving post-recurrence TACE, the median OS was significantly higher in responders than in non-responders (log-rank test: *p* = 0.0120; Figure 6K). Furthermore, TACE non-responders exhibited shorter disease-free survival compared to responders (Supplementary Figures S7C-E). These findings underscored the potential of the HCCEvoSig model as a valuable tool for predicting TACE response and guiding personalized treatment strategies in patients with HCC.

### Construction and validation of a prognostic nomogram for HCC survival prediction

**FIGURE 7:**
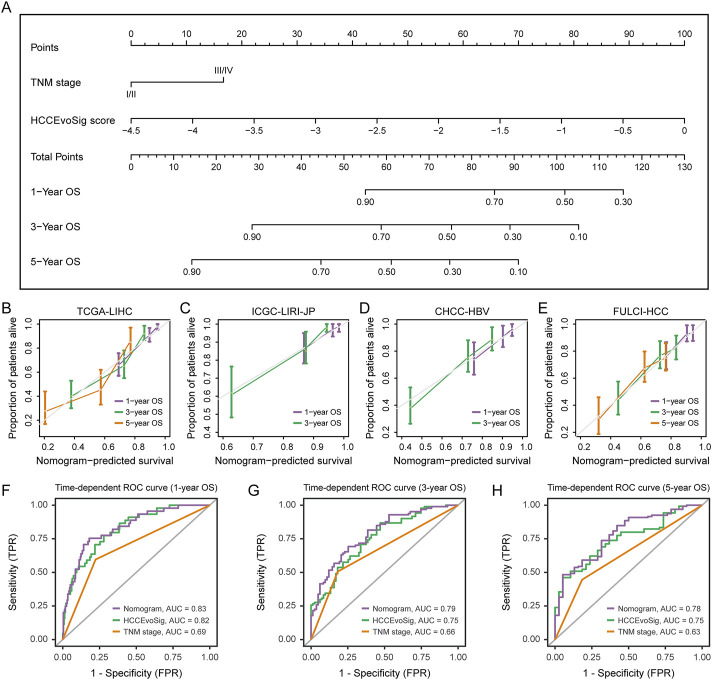
Nomogram Construction and Performance Evaluation. **(A)** Nomogram constructed based on multivariate analysis in the TCGA-LIHC cohort to predict 1-, 3-, and 5-year survival probabilities. **(B–E)** Calibration curves demonstrating the performance of the nomogram in predicting 1-, 3-, and 5-year survival in the TCGA-LIHC **(B)**, ICGC-LIRI-JP **(C)**, CHCC-HBV **(D)**, and FULCI-HCC **(E)** cohorts. The dotted line represents the ideal nomogram, while the violet, green, and pink solid lines represent the observed nomograms for 1-, 3-, and 5-year survival predictions, respectively. **(F–H)** Time-dependent receiver operating characteristic (ROC) curves evaluating the sensitivity and specificity of the nomogram, HCCEvoSig, and TNM stage in predicting 1- **(F)**, 3- **(G)**, and 5-year **(H)** survival in the TCGA-LIHC cohort.

To provide clinicians with a quantitative model for predicting the survival probability of individual HCC patients, we constructed a nomogram that integrated both HCCEvoSig and clinicopathological risk factors to estimate 1-, 3-, and 5-year outcomes. HCCEvoSig and TNM stage, both of which demonstrated independent prognostic capacity across multiple cohorts according to multivariate analysis (Supplementary Table S11, 12, 15), were incorporated into the model (Figure 7A). Calibration plots for the 1-, 3-, and 5-year survival rates indicated that the outcomes predicted by the nomogram closely approximated actual survival in TCGA-LIHC training cohort (Figure 7B) and three validation cohorts (Figures 7C–E; ICGC-LIRI-JP, CHCC-HBV, and FULCI-HCC cohorts). The discriminative ability of the nomogram (C-index: 0.76 [0.71–0.81]; 0.79 [0.72–0.86]; 0.72 [0.65–0.79]; and 0.70 [0.64–0.76] for TCGA-LIHC, ICGC-LIRI-JP, CHCC-HBV, and FULCI-HCC cohorts, respectively) was stronger than that of either HCCEvoSig or tumor TNM stage alone across multiple cohorts (Supplementary Table S22). Time-dependent ROC curves further demonstrated that the specificity and sensitivity of the prognostic nomogram were superior to any single independent predictive factor for 1-year (Figure 7F; AUC: 0.83, 0.82, and 0.69 for nomogram, HCCEvoSig, and TNM stage, respectively), 3-year (Figure 7G; AUC: 0.79, 0.75, and 0.66 for nomogram, HCCEvoSig, and TNM stage, respectively) and 5-year (Figure 7H; AUC: 0.78, 0.75, and 0.63 for nomogram, HCCEvoSig, and TNM stage, respectively) survival. These results were also validated in three independent cohorts (Supplementary Figure S8; Supplementary Table S22). Taken together, the combined nomogram based on HCCEvoSig and TNM stage enhanced survival prediction compared to the use of either prognostic factor alone.

## Discussion

Intra-tumoral heterogeneity is a common characteristic of solid malignancies and is thought to be evolutionarily selected to drive tumor cell fitness and survival (Black and McGranahan, 2021). Molecular signatures derived from single biopsies (Kim et al., 2012; Villa et al., 2016; Sun et al., 2024), based on high-throughput expression profiles of HCC tumors and/or adjacent non-tumor tissues, often overlook the regional transcriptional heterogeneity within a single tumor that may contribute to evolutionary fitness. In this study, we quantified transcriptomic heterogeneity using multiregional HCC expression profiles, and found that genes exhibiting high intra- and inter-tumor expression dynamics served as a concentrated reservoir of prognostic information. From these genes, we defined a signature, HCCEvoSig, consisting of 11 core genes, which demonstrated prognostic reproducibility and generalizability across multiple HCC cohorts and commercial profiling platforms, suggesting that HCCEvoSig recapitulated the different stages of tumor evolution observed in a large HCC cohort across a spectrum of clinical stages. To consolidate the results, we conducted multiregional single-cell transcriptomic analysis, revealing that HCCEvoSig genes demonstrated dynamic transcriptomic changes regarding intra-tumor and inter-tumor contexts, and importantly, exhibited geospatially gradual transitions from non-tumor tissue to tumor border, and tumor core, and from non-malignant to malignant epithelial cells, suggesting that they underpinned the evolution of HCC.

Since HCCEvoSig was derived from HCC prognostic indicators, we evaluated its discrimination ability and prognostic accuracy by comparing it to established multigene expression signatures. The results demonstrated that HCCEvoSig outperformed the other signatures, with only one gene (*SERPINE1*) overlapping with the established signatures (Supplementary Table S6). Additionally, we investigated whether the core genes of HCCEvoSig were included in the CancerLivER database (Kaur et al., 2020), which comprises over 594 liver cancer biomarkers, and found that four of 11 genes (*ADH4*, *CYP2C9*, *SPP1* and *SERPINE1*) were included in the database. Furthermore, we explored the presence of HCCEvoSig core genes in scientific literature using the PubMed database. The results indicated low co-occurrences of the targeted genes (components of HCCEvoSig mentioned in article titles) with hepatocellular carcinoma (mentioned in article titles or abstracts) in scientific literature (Supplementary Table S6). These findings suggested that, although prior studies tended to select EvoGenes (Figure 1E, Supplementary Table S1N), more than half of the HCCEvoSig genes have not been reported as biomarkers for HCC. Notably, a recent milestone study using single-cell sequencing analysis found that *SPP1* expression patterns follow hierarchical relationships of tumor cell branching evolution, positioning *SPP1* as a candidate regulator of tumor evolution in response to treatment (Ma et al., 2021). Supporting these findings, our results showed that *SPP1* expression exhibited dynamic changes, with a sustained increase from non-tumor tissue to tumor border, and tumor core. Importantly, compare to non-malignant EPIs, malignant EPIs exhibited upregulation of *SPP1*. Further *in vitro* and *in vivo* experimental analyses are warranted to functionally validate the genes within HCCEvoSig that exhibit intra-tumor transcriptional dynamics and drive tumor cell biodiversity.

Currently, clinical classification systems for HCC, including the AJCC TNM system, CLIP and BCLC staging, are employed to evaluate patient outcomes and provide guidelines for intervention. However, these systems cannot meet additional clinical requirements, such as drug response prediction (Vogel et al., 2022; Brown et al., 2023). The HCCEvoSig we developed demonstrated superior performance in prognostic prediction compared to these systems. Furthermore, HCCEvoSig was significantly correlated with the infiltration abundance of immune cells and the expression levels of immune checkpoint molecules. Consequently, we investigated its ability to predict outcomes of immunotherapy. The TIDE and IPS results indicated that the model held potential predictive value for assessing the efficacy of ICI therapy. Additionally, this model was capable of assigning cases into responsive and non-responsive subtypes in two retrospective cohorts from independent centers, which exhibited marked differences in both procedure and patient population, thereby underscoring robustness of this model.

In summary, our results revealed that genes exhibiting high intra- and inter-tumor expression heterogeneity were significantly enriched for HCC prognostic information. Utilized a machine-learning algorithm, we developed and validated a prediction signature, HCCEvoSig. Multiregional single-cell transcriptomic data validated that HCCEvoSig genes exhibited spatial dynamics and gradual changes from non-tumor tissue to tumor border, then to tumor core, suggesting that these genes underpinned the evolution of HCC. A higher HCCEvoSig risk score was significantly associated with adverse tumor features and patient mortality, which was validated across multiple cohorts and profiling platforms, demonstrating robust prognostic significance. Notably, HCCEvoSig also exhibited predictive utility for responses to immunotherapy and TACE. However, it is important to note that our results were based on multi-independent datasets, all of which were retrospective cohorts. Therefore, further refinement of the HCCEvoSig model and the integrated nomogram is necessary in a prospective study involving a large cohort with multiregional tumor samples.

## Data Availability

The original contributions presented in the study are included in the article/Supplementary Material, further inquiries can be directed to the corresponding authors.

## References

[B1] Black J. R. M. McGranahan N. (2021). Genetic and non-genetic clonal diversity in cancer evolution. Nat. Rev. Cancer 21 (6), 379–392. 10.1038/s41568-021-00336-2 33727690

[B2] Brown Z. J. Tsilimigras D. I. Ruff S. M. Mohseni A. Kamel I. R. Cloyd J. M. (2023). Management of hepatocellular carcinoma: a review. JAMA Surg. 158 (4), 410–420. 10.1001/jamasurg.2022.7989 36790767

[B3] Calderaro J. Couchy G. Imbeaud S. Amaddeo G. Letouze E. Blanc J. F. (2017). Histological subtypes of hepatocellular carcinoma are related to gene mutations and molecular tumour classification. J. Hepatol. 67 (4), 727–738. 10.1016/j.jhep.2017.05.014 28532995

[B4] Cancer Genome Atlas Research Network (2017). Electronic address wbe, cancer genome atlas research N. Comprehensive and integrative genomic characterization of hepatocellular carcinoma. Cell 169 (7), 1327–41 e23. 10.1016/j.cell.2017.05.046 28622513 PMC5680778

[B5] Candia J. Bayarsaikhan E. Tandon M. Budhu A. Forgues M. Tovuu L. O. (2020). The genomic landscape of Mongolian hepatocellular carcinoma. Nat. Commun. 11 (1), 4383. 10.1038/s41467-020-18186-1 32873799 PMC7462863

[B6] Charoentong P. Finotello F. Angelova M. Mayer C. Efremova M. Rieder D. (2017). Pan-cancer immunogenomic analyses reveal Genotype-Immunophenotype relationships and predictors of response to checkpoint blockade. Cell Rep. 18 (1), 248–262. 10.1016/j.celrep.2016.12.019 28052254

[B7] Chen B. Khodadoust M. S. Liu C. L. Newman A. M. Alizadeh A. A. (2018). Profiling tumor infiltrating immune cells with CIBERSORT. Methods Mol. Biol. 1711, 243–259. 10.1007/978-1-4939-7493-1_12 29344893 PMC5895181

[B8] Chen J. Kaya N. A. Zhang Y. Kendarsari R. I. Sekar K. Lee C. S. (2024). A multimodal atlas of hepatocellular carcinoma reveals convergent evolutionary paths and 'bad apple' effect on clinical trajectory. J. Hepatol. 81 (4), 667–678. 10.1016/j.jhep.2024.05.017 38782118

[B9] Craig A. J. von Felden J. Garcia-Lezana T. Sarcognato S. Villanueva A. (2020). Tumour evolution in hepatocellular carcinoma. Nat. Rev. Gastroenterol. Hepatol. 17 (3), 139–152. 10.1038/s41575-019-0229-4 31792430

[B10] Duseja A. (2014). Staging of hepatocellular carcinoma. J. Clin. Exp. Hepatol. 4 (Suppl. 3), S74–S79. 10.1016/j.jceh.2014.03.045 25755615 PMC4284240

[B11] Fako V. Martin S. P. Pomyen Y. Budhu A. Chaisaingmongkol J. Franck S. (2019). Gene signature predictive of hepatocellular carcinoma patient response to transarterial chemoembolization. Int. J. Biol. Sci. 15 (12), 2654–2663. 10.7150/ijbs.39534 31754337 PMC6854367

[B12] Fu J. Li K. Zhang W. Wan C. Zhang J. Jiang P. (2020). Large-scale public data reuse to model immunotherapy response and resistance. Genome Med. 12 (1), 21. 10.1186/s13073-020-0721-z 32102694 PMC7045518

[B13] Fujimoto A. Furuta M. Totoki Y. Tsunoda T. Kato M. Shiraishi Y. (2016). Whole-genome mutational landscape and characterization of noncoding and structural mutations in liver cancer. Nat. Genet. 48 (5), 500–509. 10.1038/ng.3547 27064257

[B14] Ganesan P. Kulik L. M. (2023). Hepatocellular carcinoma: new developments. Clin. Liver Dis. 27 (1), 85–102. 10.1016/j.cld.2022.08.004 36400469

[B15] Gao Q. Zhu H. Dong L. Shi W. Chen R. Song Z. (2019). Integrated Proteogenomic characterization of HBV-related hepatocellular carcinoma. Cell 179 (2), 1240–77 e22. 10.1016/j.cell.2019.10.038 31730861

[B16] Kaur H. Bhalla S. Kaur D. Raghava G. P. (2020). CancerLivER: a database of liver cancer gene expression resources and biomarkers. Database (Oxford) 2020, 2020. 10.1093/database/baaa012 PMC7061090 32147717

[B17] Kim S. M. Leem S. H. Chu I. S. Park Y. Y. Kim S. C. Kim S. B. (2012). Sixty-five gene-based risk score classifier predicts overall survival in hepatocellular carcinoma. Hepatology 55 (5), 1443–1452. 10.1002/hep.24813 22105560 PMC4060518

[B18] Lee J. S. Heo J. Libbrecht L. Chu I. S. Kaposi-Novak P. Calvisi D. F. (2006). A novel prognostic subtype of human hepatocellular carcinoma derived from hepatic progenitor cells. Nat. Med. 12 (4), 410–416. 10.1038/nm1377 16532004

[B19] Losic B. Craig A. J. Villacorta-Martin C. Martins-Filho S. N. Akers N. Chen X. (2020). Intratumoral heterogeneity and clonal evolution in liver cancer. Nat. Commun. 11 (1), 291. 10.1038/s41467-019-14050-z 31941899 PMC6962317

[B20] Luo S. Jia Y. Zhang Y. Zhang X. (2023). A transcriptomic intratumour heterogeneity-free signature overcomes sampling bias in prognostic risk classification for hepatocellular carcinoma. JHEP Rep. 5 (6), 100754. 10.1016/j.jhepr.2023.100754 37234275 PMC10206488

[B21] Ma L. Hernandez M. O. Zhao Y. Mehta M. Tran B. Kelly M. (2019). Tumor cell biodiversity drives microenvironmental reprogramming in liver cancer. Cancer Cell 36 (4), 418–430. 10.1016/j.ccell.2019.08.007 31588021 PMC6801104

[B22] Ma L. Wang L. Khatib S. A. Chang C. W. Heinrich S. Dominguez D. A. (2021). Single-cell atlas of tumor cell evolution in response to therapy in hepatocellular carcinoma and intrahepatic cholangiocarcinoma. J. Hepatol. 75 (6), 1397–1408. 10.1016/j.jhep.2021.06.028 34216724 PMC8604764

[B23] Ma L. Heinrich S. Wang L. Keggenhoff F. L. Khatib S. Forgues M. (2022). Multiregional single-cell dissection of tumor and immune cells reveals stable lock-and-key features in liver cancer. Nat. Commun. 13 (1), 7533. 10.1038/s41467-022-35291-5 36476645 PMC9729309

[B24] Pinato D. J. Murray S. M. Forner A. Kaneko T. Fessas P. Toniutto P. (2021). Trans-arterial chemoembolization as a loco-regional inducer of immunogenic cell death in hepatocellular carcinoma: implications for immunotherapy. J. Immunother. Cancer 9 (9). 10.1136/jitc-2021-003311 PMC8487214 34593621

[B25] Ren Z. Wang Y. Jiang D. Liu Y. Yang X. Wang T. (2025). PD1(+) Treg cell remodeling promotes immune homeostasis within peripheral blood and tumor microenvironment after microparticles-transarterial chemoembolization in hepatocellular carcinoma. Cancer Immunol. Immunother. 74 (3), 109. 10.1007/s00262-025-03962-z 39937280 PMC11822157

[B26] Roessler S. Jia H. L. Budhu A. Forgues M. Ye Q. H. Lee J. S. (2010). A unique metastasis gene signature enables prediction of tumor relapse in early-stage hepatocellular carcinoma patients. Cancer Res. 70 (24), 10202–10212. 10.1158/0008-5472.CAN-10-2607 21159642 PMC3064515

[B27] Satija R. Farrell J. A. Gennert D. Schier A. F. Regev A. (2015). Spatial reconstruction of single-cell gene expression data. Nat. Biotechnol. 33 (5), 495–502. 10.1038/nbt.3192 25867923 PMC4430369

[B28] Shen Y. C. Hsu C. L. Jeng Y. M. Ho M. C. Ho C. M. Yeh C. P. (2020). Reliability of a single-region sample to evaluate tumor immune microenvironment in hepatocellular carcinoma. J. Hepatol. 72 (3), 489–497. 10.1016/j.jhep.2019.09.032 31634533

[B29] Shi M. Lu L. G. Fang W. Q. Guo R. P. Chen M. S. Li Y. (2013). Roles played by chemolipiodolization and embolization in chemoembolization for hepatocellular carcinoma: single-blind, randomized trial. J. Natl. Cancer Inst. 105 (1), 59–68. 10.1093/jnci/djs464 23150720

[B30] Shi L. Zhang Y. Feng L. Wang L. Rong W. Wu F. (2017). Multi-omics study revealing the complexity and spatial heterogeneity of tumor-infiltrating lymphocytes in primary liver carcinoma. Oncotarget 8 (21), 34844–34857. 10.18632/oncotarget.16758 28422742 PMC5471016

[B31] Singal A. G. Kanwal F. Llovet J. M. (2023). Global trends in hepatocellular carcinoma epidemiology: implications for screening, prevention and therapy. Nat. Rev. Clin. Oncol. 20 (12), 864–884. 10.1038/s41571-023-00825-3 37884736

[B32] Sun Z. Liu H. Zhao Q. Li J. H. Peng S. F. Zhang Z. (2024). Immune-related cell death index and its application for hepatocellular carcinoma. NPJ Precis. Oncol. 8 (1), 194. 10.1038/s41698-024-00693-9 39245753 PMC11381516

[B33] Sung H. Ferlay J. Siegel R. L. Laversanne M. Soerjomataram I. Jemal A. (2021). Global cancer statistics 2020: GLOBOCAN estimates of Incidence and mortality worldwide for 36 cancers in 185 Countries. CA Cancer J. Clin. 71 (3), 209–249. 10.3322/caac.21660 33538338

[B34] Thorsson V. Gibbs D. L. Brown S. D. Wolf D. Bortone D. S. Ou Yang T. H. (2018). The immune landscape of cancer. Immunity 48 (4), 812–30 e14. 10.1016/j.immuni.2018.03.023 29628290 PMC5982584

[B35] Villa E. Critelli R. Lei B. Marzocchi G. Camma C. Giannelli G. (2016). Neoangiogenesis-related genes are hallmarks of fast-growing hepatocellular carcinomas and worst survival. Results from a prospective study. Gut 65 (5), 861–869. 10.1136/gutjnl-2014-308483 25666192

[B36] Vogel A. Meyer T. Sapisochin G. Salem R. Saborowski A. (2022). Hepatocellular carcinoma. Lancet 400 (10360), 1345–1362. 10.1016/S0140-6736(22)01200-4 36084663

[B37] Yang C. Zhang S. Cheng Z. Liu Z. Zhang L. Jiang K. (2022). Multi-region sequencing with spatial information enables accurate heterogeneity estimation and risk stratification in liver cancer. Genome Med. 14 (1), 142. 10.1186/s13073-022-01143-6 36527145 PMC9758830

[B38] Zhai W. Lai H. Kaya N. A. Chen J. Yang H. Lu B. (2022). Dynamic phenotypic heterogeneity and the evolution of multiple RNA subtypes in hepatocellular carcinoma: the PLANET study. Natl. Sci. Rev. 9 (3), nwab192. 10.1093/nsr/nwab192 35382356 PMC8973408

