# Supplementary material for: Spatial heterogeneity reveals an evolutionary signature predicting therapeutic response and clinical outcomes in hepatocellular carcinoma
Source: Front Bioinform. 2025 Aug 18;5:1669236. doi: 10.3389/fbinf.2025.1669236 (PMC12399655; doi:10.3389/fbinf.2025.1669236)
Supplement: Supplementary file 1 [file Presentation1.pdf]

Supplementary Materials

Spatial Heterogeneity Reveals an Evolutionary Signature Predicting Therapeutic Response and Clinical Outcomes in Hepatocellular Carcinoma

Shangyi Luo, Li Liu, Yang Sun, Jian Shi, Yajing Zhang

Table of contents

Supplementary methods ……………………………………………………………….2

Supplementary figures ………………………………………………………………9

Supplementary tables ………………………………………………………………19

Supplementary methods

HCC cohorts with multiregional transcriptome data

For the MultiRRnaSeq1 cohort, the original article includes 51 tumor samples from 14 HCC patients. However, the RNA-Seq FASTQ files for two tumor samples from one patient were not publicly available due to lack of patient specific deposition consent. Additionally, two patients, including three and four tumor samples respectively, were profiled solely by single-cell RNA-sequencing. Consequently, bulk RNA sequencing data from 42 tumor samples of 11 patients were used in this study. The relevant RNA-Seq FASTQ files were downloaded from the ArrayExpress database (accession number: E-MTAB-5905; https://www.ebi.ac.uk/arrayexpress/). Raw sequencing reads were mapped to the human reference genome (GRCh37/NCBI) using Bowtie. Transcript expression was quantified using the RSEM package with RefSeq annotation to generate counts and transcript per million (TPM) expression values. Genes with an expression value of at least 1 TPM in at least 20% (9/42) of tumor samples were retained for downstream analyses.

For the MultiRRnaSeq2 cohort, raw RNA sequencing data was unavailable, thus we obtained preprocessed, normalized expression values (TPM) from Zenodo through the following link: https://zenodo.org/record/7336311#.Y-snVnZByPo.

Expression profiles for the MultiRRnaSeq3 cohort were downloaded from the NCBI's Gene Expression Omnibus (GEO) database in the form of raw fragment counts (accession number: GSE136711).

For the MultiRArray cohort, raw CEL files of Agilent 4×44 K Whole Human Genome Oligo microarrays were also downloaded from the GEO database (accession number: GSE92528).

HCC cohorts with survival information and gene expression data

For the TCGA-LIHC cohort, transcriptome data of 373 liver cancer specimens (including 371 primary and 2 recurrent tumor specimens) and 50 corresponding normal specimens from 371 patients were downloaded from the Broad Institute's GDAC Firehose (https://gdac.broadinstitute.org). The "Scaled_estimate" was used to calculate

TPM by multiplying 1,000,000. We only included 371 primary tumors and 50 corresponding normal specimens from 371 liver cancer patients in our study. For the 371 patients, patients who suffered from other types of cancers (e.g. prostate adenocarcinoma) prior to diagnosis of HCC (8 patients), who received neoadjuvant therapy (2 patients), whose pathological diagnosis other than HCC (e.g. intrahepatic cholangiocarcinoma (ICC), and combined hepatocellular cholangiocarcinoma (cHCC/CC)) (11 patients), who had inferior specimens (e.g. not representative of a hepatocellular case) (2 patients), and who were recurrent HCC (1 patient) were excluded from further analysis according to the TCGA sample quality and clinical annotations file from the TCGA Pan-Caner Atlas (https://gdc.cancer.gov/about-data/publications/). To rule out non-neoplastic causes of death, including heart failure, postoperative infection and bleeding, and to remove patients with limited follow-up periods, we further excluded 24 patients (17 of 24 (71%) patients were still alive) whose follow-up duration or OS less than 1 month, including 1 patient with missing follow-up information (the OS time was unavailable), 5 patients with 0-day follow-up period, 14 patients with less than 20-day follow-up period and 4 patients with 20 to 27-day follow-up period. The remaining 323 HCC samples and 42 paired normal samples were designated as the training cohort. Clinicopathological characteristics and somatic mutation profiles of HCC were also downloaded from the TCGA Pan-Caner Atlas. An expression filter was applied to retain genes with an expression value of at least 1 TPM in at least 20% (65/323) of tumor samples, before obtaining normalized count values for filtered genes.

For the ICGC-LIRI-JP cohort, raw RNA-Seq count data for 243 liver cancer specimens and 202 corresponding normal specimens from 232 patients were downloaded from the ICGC portal (https://dcc.icgc.org/). Clinicopathological characteristics and somatic mutation profiles were obtained from the same source. Forty liver cancer specimens were excluded from further analysis, including 3 with metastatic tumor, 30 with ICC or cHCC/CC, and 7 duplicated samples with low tumor cell percentages. The remaining 203 HCC samples and 175 paired normal samples were designated as the internal validation cohort. Transcriptome data with annotated gene symbols were filtered to

retain genes with an expression value of at least 1 read in at least 20% (41/203) of HCC samples.

RNA-Seq data from 159 paired tumor-normal samples of the Chinese HCC patients with hepatitis B virus infection (CHCC-HBV cohort) were downloaded from the National Omics Data Encyclopedia (accession number: OEP000321, https://www.biosino.org/node). The clinicopathological characteristics and somatic mutation data for the CHCC-HBV cohort were obtained from the Supplementary Materials of reference (1).

Gene expression and clinical data from the National Cancer Institute (NCI-HCC), Fudan University Liver Cancer Institute (FULCI-HCC) and Mongolian (Mongolian-HCC) HCC cohorts, as reported in previous studies (2-4), were acquired from the NCBI's GEO database (accession numbers: GSE1898, GSE4024, GSE14520 and GSE144269).

For the NCI-HCC cohort (n = 140 samples), gene expression profiling was performed by the Laboratory of Experimental Carcinogenesis (LEC) and analyzed using NCI's Human Array-Ready Oligo Set microarray platform (Platform: GPL1528). As raw data was unavailable, preprocessed, normalized expression values were obtained from the GEO database with accession numbers GSE1898 and GSE4024. In this dataset, 28 HCC patients lacking follow-up information were excluded from survival analysis.

Gene expression profiling of 247 HCC samples from the FULCI-HCC cohort, originating from the Liver Cancer Institute of Fudan University, is publicly available at NCBI's GEO with accession number GSE14520. Within this cohort, 225 samples were analyzed using the Affymetrix HT Human Genome U133A Array (Platform: GPL3921), and 22 samples were analyzed using the Affymetrix Human Genome U133A 2.0 Array (Platform: GPL571). In the current study, 225 HCC samples paired with 214 normal samples from the GPL571 platform were used for downstream analysis. Disease-free survival, overall survival, and cause of death data were available for 221 patients.

For the Mongolian-HCC cohort, raw RNA-Seq count data for 70 paired HCC tumor-normal samples were downloaded from the NCBI's GEO database with accession number GSE144269. Matched clinicopathological characteristics and somatic mutation

data were obtained from the GitHub repository (https://github.com/juliancandia/MongolianHCC).

HCC cohort with tumor doubling times and gene expression data

Gene expression data and HCC doubling times which were calculated from the imaging data of the GSE54236 cohort, were obtained from the NCBI's GEO database.

Normalization of gene expression data

For the RNA-Seq data, a variance stabilizing transformation (VST) was applied to counts from filtered genes using the R package DESeq2, assuming a negative binomial distribution for count values, to yield homoscedastic and library size-normalized count values. For the Agilent and Affymetrix data, the quantile normalization method from the R package limma and Robust Multi-array Average (RMA) method from the R package affy were employed, from which we got the background-corrected, quantile-normalized and log base 2 transformed gene expression matrix. Control probes and probes that don’t appear to be expressed were filtered out. When multiple probes corresponded to the same gene, the probe with the highest normalized intensity averaged over all samples was used.

Collection of previously published HCC prognostic gene expression signatures

Fifteen previously published HCC prognostic gene expression signatures were compiled. Each of these signatures is defined by a specific formula, detailed in Table S3. These signatures were constructed using gene expression levels and regression coefficients derived from univariate Cox, multivariate Cox, or least absolute shrinkage and selection operator (LASSO) Cox regression analyses. Signature risk scores were calculated using a linear combination of each gene's expression value multiplied by its corresponding coefficient using the following formula: Signature Risk Score = $\sum_{i=1}^{n}((coefficient\ of\ gene\ i) \times (expression\ value\ of\ gene\ i))$. The signature-specific formula was applied to different expression matrices to obtain a risk score for

each sample. For each of the 15 signatures, samples were dichotomized into high- or low-risk groups based on the median risk score within each cohort.

Gene heterogeneity scores and quadrants

Inter-tumor and intra-tumor gene heterogeneity scores were calculated using multi-regional gene expression data, including only patients with more than two multi-regional samples. In the MultiRRnaSeq1 cohort, sample H9.c was excluded from downstream analyses because it originated from a separate tumor nodule of patient P09. Additionally, the technical replicate of sample H2.a was also removed. The inter-tumor gene heterogeneity score for each gene was calculated by randomly sampling one region per patient and calculating the standard deviation across the resulting single-sample cohort. This process was repeated ten times, and the average score across iterations was used as the final inter-tumor heterogeneity score. Similarly, the intra-tumor gene heterogeneity score for each gene was calculated by determining the standard deviation of expression values across tumor regions within each patient and then averaging these standard deviations across all patients in the cohort. Gene heterogeneity quadrants (Q1-Q4 quadrants) were defined by partitioning genes based on their inter-tumor and intra-tumor heterogeneity scores, using the 75th percentile as the cutoff.

Gene set enrichment analysis

Gene set enrichment analysis was performed on EvoGenes to identify enriched Gene Ontology (GO) terms in the biological process and KEGG pathways. The enrichGO and enrichKEGG functions from the R package clusterProfiler were used with default parameters. A Benjamini-Hochberg adjusted p value < 0.05 was considered statistically significant.

scRNA-seq data preprocessing and quality control

Quality control and downstream analyses were conducted using the Seurat package in R. Genes detected in fewer than ten cells and cells with low-complexity libraries

(defined as those with detected transcripts aligned to fewer than 500 genes) were filtered out and excluded from subsequent analyses. Additionally, cells with mitochondrial RNA percentages greater than 20% were removed. After the initial clustering, likely cell doublets were eliminated from all clusters based on the following criteria: (1) Library complexity: cells considered outliers in terms of library complexity, specifically those with more than 6,000 expressed genes or over 50,000 unique molecular identifiers (UMIs). (2) Cluster distribution: doublets or multiplets that formed distinct clusters with hybrid expression features and exhibited an aberrantly high gene count. (3) Cluster marker gene expression: cells within a cluster expressed markers from distinct lineages (e.g., cells in the T cell cluster exhibiting expression of epithelial cell markers). We carefully reviewed canonical marker gene expression on UMAP and tSNE plots, and repeated the steps above a couple of times to ensure that most barcodes associated with cell doublets were excluded. Following quality filtering, a total of 110,817 cells were retained for downstream analysis.

Dimensionality reduction, clustering, and annotation of cell types

Filtered single cells were analyzed using Seurat's standard analysis pipeline. First, the filtered gene-cell matrix was normalized for sequencing depth by dividing the total number of unique molecular identifiers (UMIs) for each cell and then transformed to a log scale using the NormalizeData and ScaleData functions. Highly variable genes were identified using the FindVariableGenes function with default parameters. Dimensionality reduction was performed using principal component analysis (PCA) with the RunPCA function, and the optimal number of principal components (PCs) was determined using the ElbowPlot function. Cells were then clustered using Seurat’s FindNeighbors with dimensions 1-30 and FindClusters with a resolution of 0.8. For visualization, dimensionality was further reduced using either the t-SNE or UMAP methods via the RunTSNE and RunUMAP functions, respectively. The principal components used for calculating the embeddings were the same as those used for clustering. To define major cell types, differentially expressed genes (DEGs) were identified for each cell cluster using the FindAllMarkers analysis (parameters: genes

detected in at least 25% cells, and differential expression threshold of 0.25 log fold change, using Wilcoxon rank sum test with $p < 0.05$ following Bonferroni correction). The top 20 most significant DEGs were carefully reviewed. In parallel, feature plots were generated for the top 20 DEGs and a suggested set of canonical cell-type markers, followed by a manual review process. Enrichment of these markers (e.g., *EPCAM*/*KRT8*/*KRT18* for epithelial cells, *CD3D*/*CD3E*/*CD3G* for T cells, *MS4A1*/*CD79A*/*CD19* for B cells, *COL1A1*/*COL1A2*/*DCN* for fibroblasts, *CD14*/*CD68*/*CD163* for macrophages, and *PECAM1*/*VWF*/*CDH5* for endothelial cells) in specific clusters was considered a strong indication that the clusters represented the corresponding cell types. These two approaches were combined to infer major cell types for each cell cluster based on the enrichment of marker genes and top-ranked DEGs. All dot plots were generated using the DotPlot function, all expression plots on the UMAP or tSNE projections were created with the FeaturePlot function, and all violin plots were generated using the VlnPlot function.

Reference

1. Gao Q, Zhu H, Dong L, Shi W, Chen R, Song Z, et al. Integrated Proteogenomic Characterization of HBV-Related Hepatocellular Carcinoma. Cell. 2019;179(2):561-77 e22.
2. Lee JS, Heo J, Libbrecht L, Chu IS, Kaposi-Novak P, Calvisi DF, et al. A novel prognostic subtype of human hepatocellular carcinoma derived from hepatic progenitor cells. Nat Med. 2006;12(4):410-6.
3. Roessler S, Jia HL, Budhu A, Forgues M, Ye QH, Lee JS, et al. A unique metastasis gene signature enables prediction of tumor relapse in early-stage hepatocellular carcinoma patients. Cancer Res. 2010;70(24):10202-12.
4. Candia J, Bayarsaikhan E, Tandon M, Budhu A, Forgues M, Tovuu LO, et al. The genomic landscape of Mongolian hepatocellular carcinoma. Nat Commun. 2020;11(1):4383.

Supplementary figures

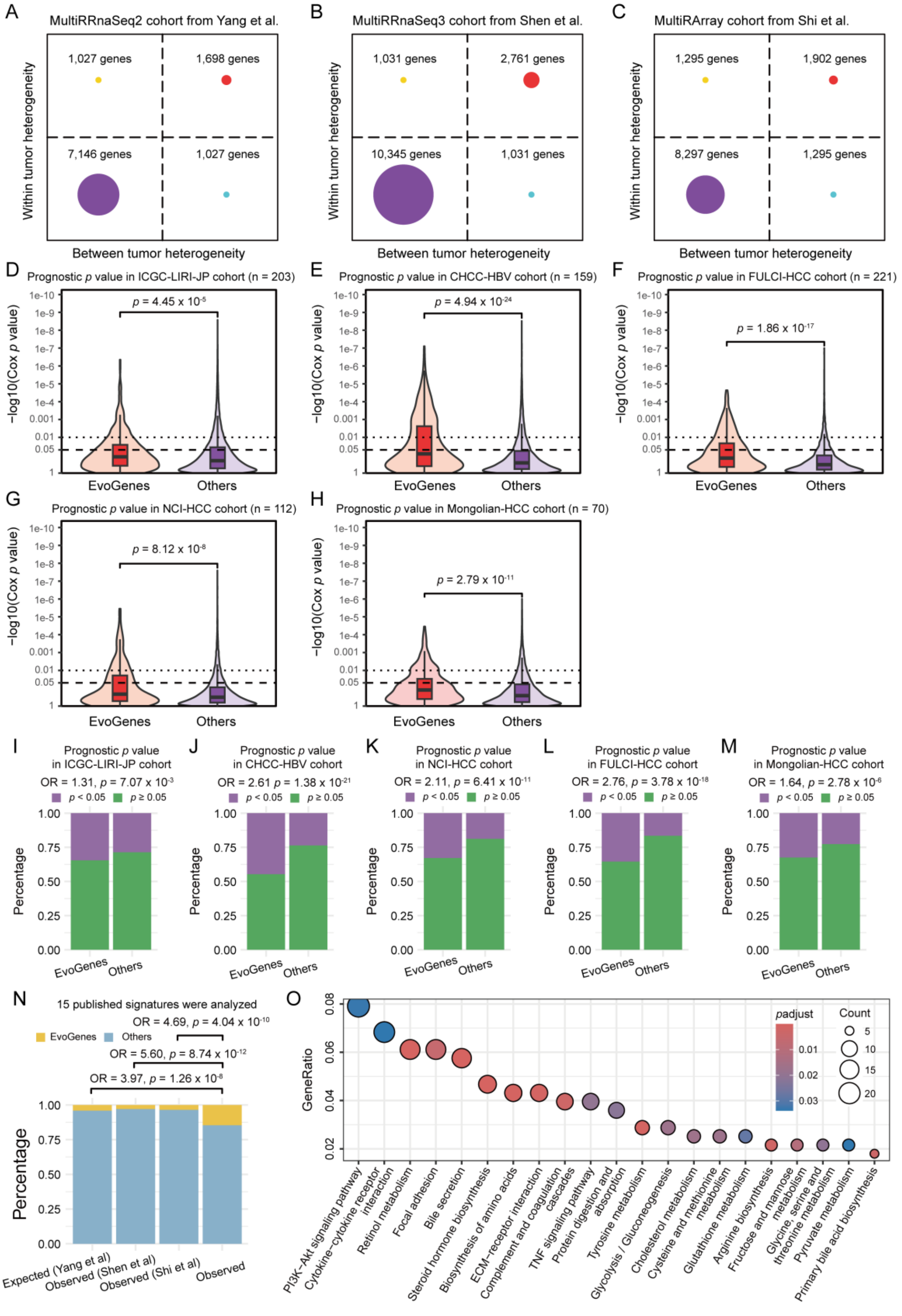

Figure S1. Gene Expression Heterogeneity and Prognostic Significance. (A-C) Gene expression inter- and intra-tumoral heterogeneity quadrants calculated using the MultiRRnaSeq2 (A), MultiRRnaSeq3 (B) and MultiRArray (C) cohorts. Quadrants

(Q1-Q4) were defined based on the 75th percentile of inter- and intra-tumoral heterogeneity scores. (D-H) Prognostic value of genes in the ICGC-LIRI-JP (D), CHCC-HBV (E), FULCI-HCC (F), NCI-HCC (G), and Mongolian-HCC (H) cohorts, assessed using Cox univariate *p* values, stratified by EvoGenes and other genes. Box plots display median values, as well as the 25th and 75th percentiles, with vertical bars spanning the 5th to 95th percentiles. Statistical significance was determined using a two-sided Wilcoxon signed-rank test. (I-M) Percentage of genes with prognostic significance (univariate Cox $p < 0.05$) in the ICGC-LIRI-JP (I), CHCC-HBV (J), FULCI-HCC (K), NCI-HCC (L), and Mongolian-HCC (M) cohorts, stratified by EvoGenes and other genes. (N) Stacked bar plot showing the percentage of EvoGenes in expected (all expressed genes in the MultiRRnaSeq2 (left), MultiRRnaSeq3 (middle), and MultiRArray (right)) versus observed (genes merged from 15 published HCC prognostic signatures) categories. Statistical significance was determined using a two-sided Fisher's exact test. (O) Bubble plot depicting KEGG enrichment results for EvoGenes. Node size represents the number of genes, and node color indicates the adjusted *p*-value for each KEGG pathway.

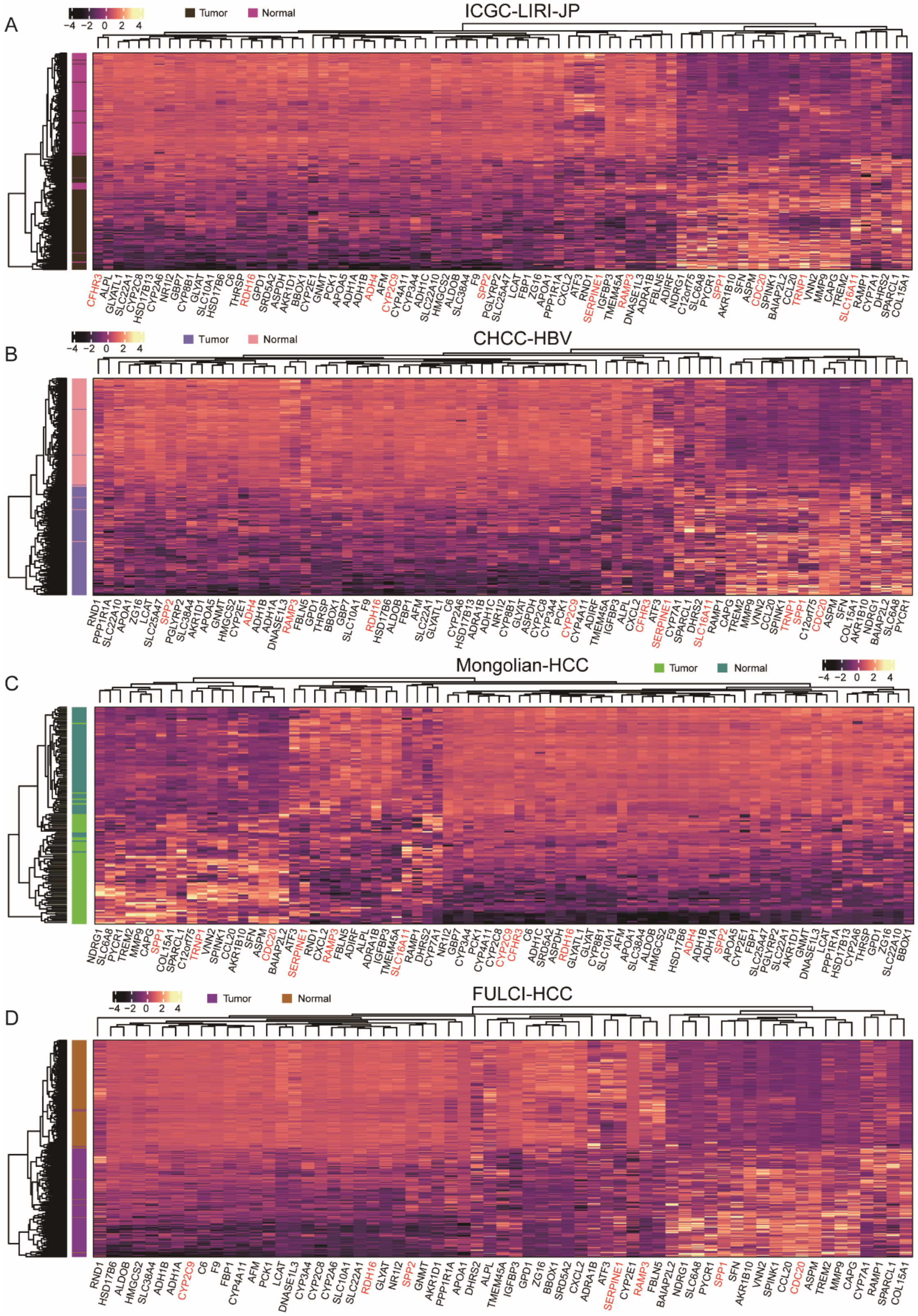

Figure S2. Expression of Candidate Prognostic Genes. (A-D) Heatmaps displaying the expression of 80 candidate reproducible prognostic genes in paired tumor and normal samples from the ICGC-LIRI-JP (A), CHCC-HBV (B), Mongolian-HCC (C), and FULCI-HCC (D) cohorts.

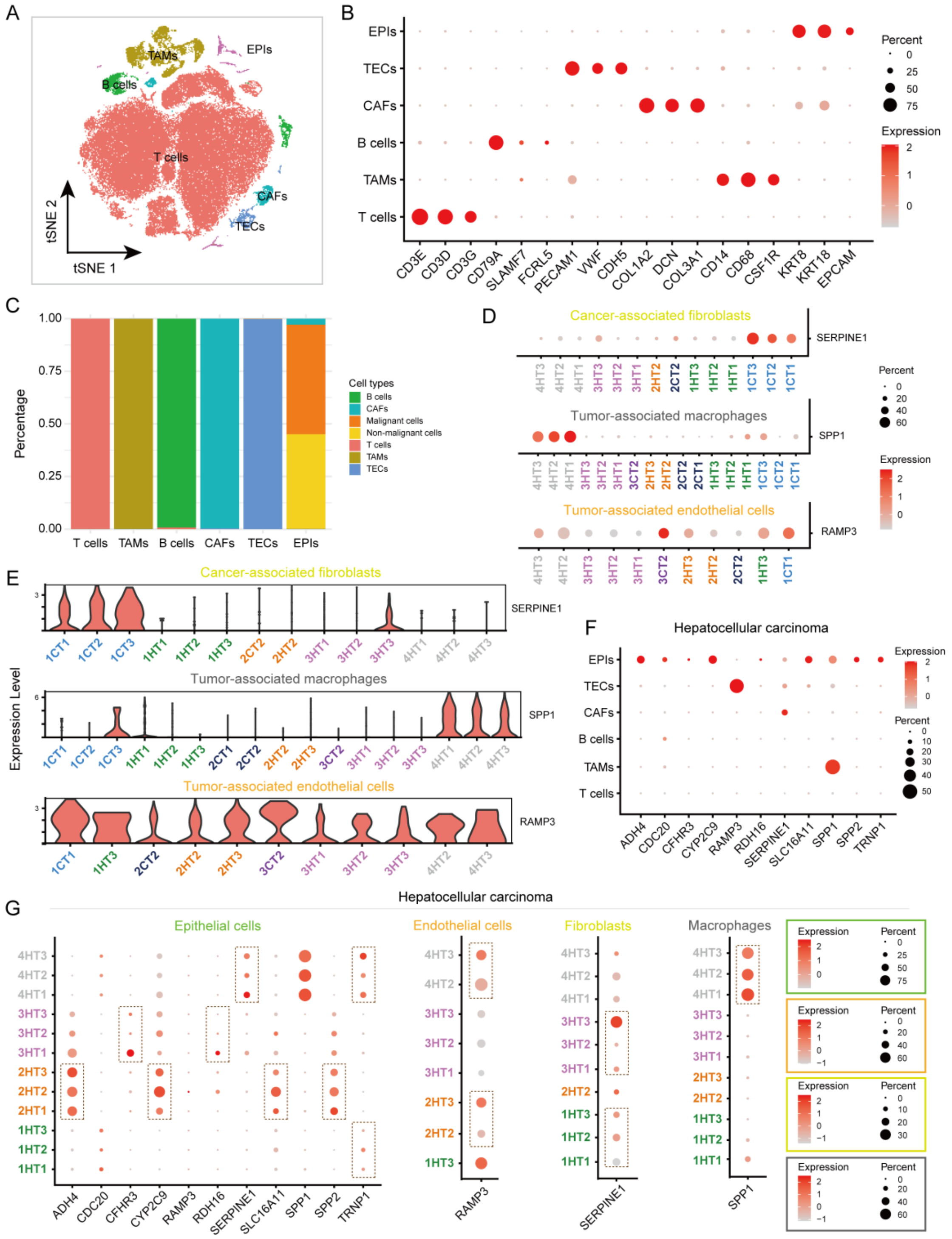

Figure S3. HCCEvoSig Genes Demonstrate Dynamic Transcriptomic Changes between Intra-tumor and Inter-tumor Contexts. (A) tSNE plot of 110,817 filtered single cells, colored by their assigned cell types: T cells, tumor-associated macrophages (TAMs), B cells, epithelial cells (EPIs), cancer-associated fibroblasts (CAFs), and tumor-associated endothelial cells (TECs). (B) Dot plot showing the expression of

marker genes for each cell type. (C) Stacked plot illustrating the consistency of cell identities identified in this study with those reported in the original study. (D) Dot plots depicting the percentage of CAFs (upper), TAMs (middle) and TECs (bottom) expressing *SERPINE1*, *SPP1* and *RAMP3* across different patients and tumor cores (indicated by the size of the circle), as well as their scaled expression levels (indicated by the color of the circle). (E) Violin plot displaying the expression levels of *SERPINE1*, *SPP1* and *RAMP3* in CAFs (upper), TAMs (middle) and TECs (bottom) across different patients and tumor cores. (F) Dot plots illustrating the percentage of each cell type expressing HCCEvoSig genes (indicated by the size of the circle) in HCC, along with their scaled expression levels (indicated by the color of the circle). (G) Dot plots illustrating the percentage of EPIs, TECs, CAFs, and TAMs expressing HCCEvoSig genes across different HCC patients and tumor cores (indicated by the size of the circle), as well as their scaled expression levels (indicated by the color of the circle).

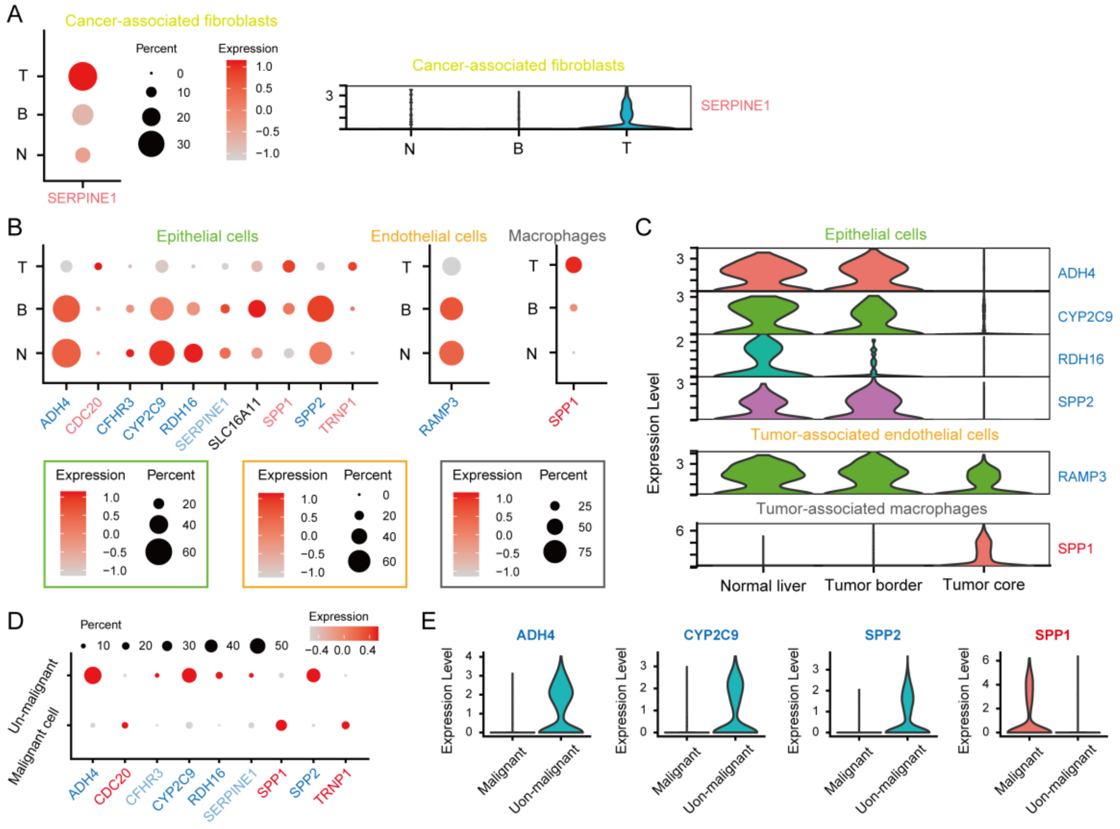

Figure S4. Geospatially Consistent Transition of HCCEvoSig Gene Expression in HCC. (A) Dot plot (left) and violin plot (right) showing the expression of *SERPINE1* in CAFs across geospatial regions: non-tumor tissue (N), tumor border (B), and tumor

core (T). *SERPINE1*, colored in light red, indicates that it did not reach the strict statistical significance threshold defined in the Methods. See Table S7 for details. (B) Dot plots depicting the expression of HCCEvoSig genes that are differentially expressed across geospatial regions in EPIs (left), TECs (middle) and TAMs (right). The size of the circle represents the percentage of cells expressing the gene in that specific region, while the color indicates the average expression of the gene. Genes marked in blue and red indicate down-regulation and up-regulation in the tumor core compared to normal tissue, consistent with bulk analysis. Light blue and light red genes did not reach the strict statistical significance threshold defined in the Methods. See Table S8 for details. (C) Violin plots for representative genes expressed in EPIs (upper), TECs (middle) and TAMs (bottom), exhibiting differential expression across geospatial regions in HCC. The color coding of the genes is consistent with that in panel B. (D) Dot plots depicting the expression of HCCEvoSig genes that are differentially expressed between malignant EPIs and non-malignant EPIs in HCC. Genes marked in blue and red indicate down-regulation and up-regulation in malignant EPIs compared to non-malignant EPIs. Light blue and light red genes did not reach the strict statistical significance threshold defined in the Methods. See Table S10 for details. (E) Violin plots of representative HCCEvoSig genes that are differentially expressed between malignant EPIs and non-malignant EPIs in HCC. The color coding of the genes is consistent with that in panel D.

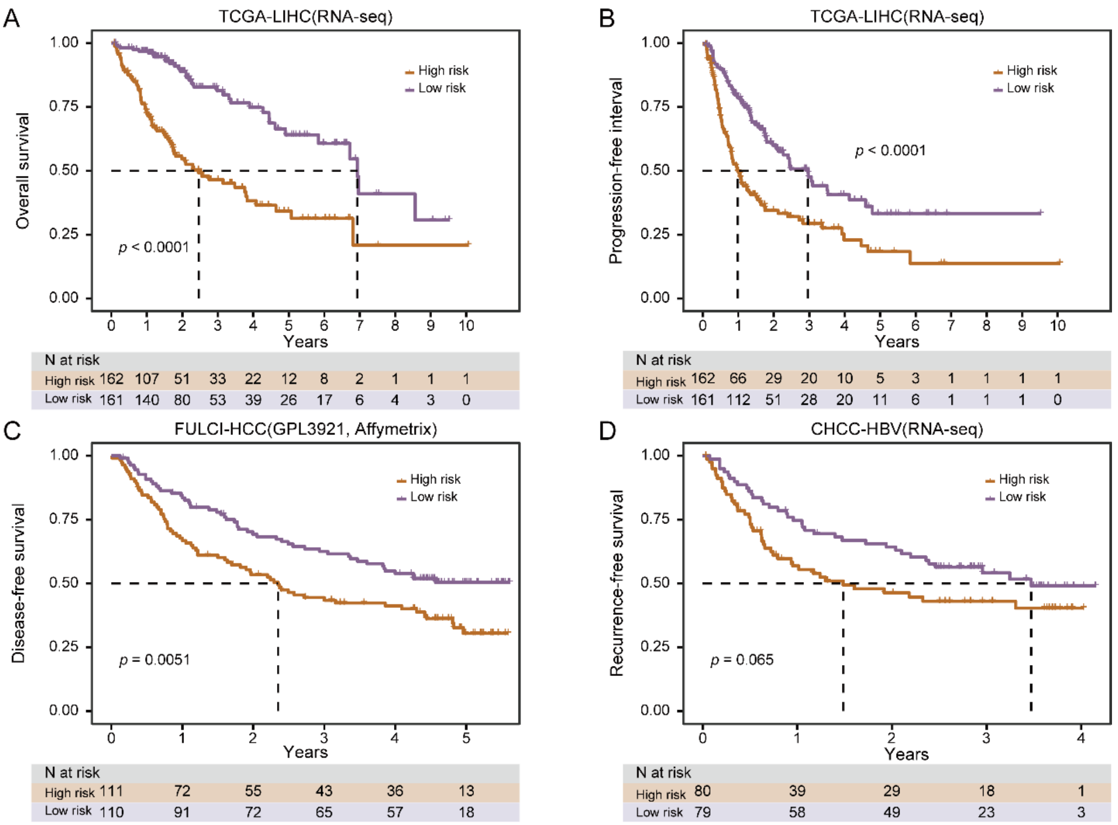

Figure S5. Robust and Reproducible Prognostic Efficacy of HCCEvoSig Across HCC Cohorts and Profiling Platforms. (A) Kaplan-Meier overall survival analysis of HCC patients in the TCGA-LIHC training cohort. (B-D) Kaplan-Meier analysis of tumor progression in HCC patients stratified by HCCEvoSig risk score, using the TCGA-LIHC training cohort (B), the independent microarray-based validation cohort FULCI-HCC (C), and the independent RNA-seq-based validation cohort CHCC-HBV (D). Patients in each dataset were divided into high- and low-risk groups based on the median HCCEvoSig risk score. The dashed lines indicate the subgroup-specific median overall survival (A), progression-free interval (B), disease-free survival (C) and recurrence-free survival (D). The number of patients at risk in each group at various time points and the log-rank *p* value are indicated for each graph.

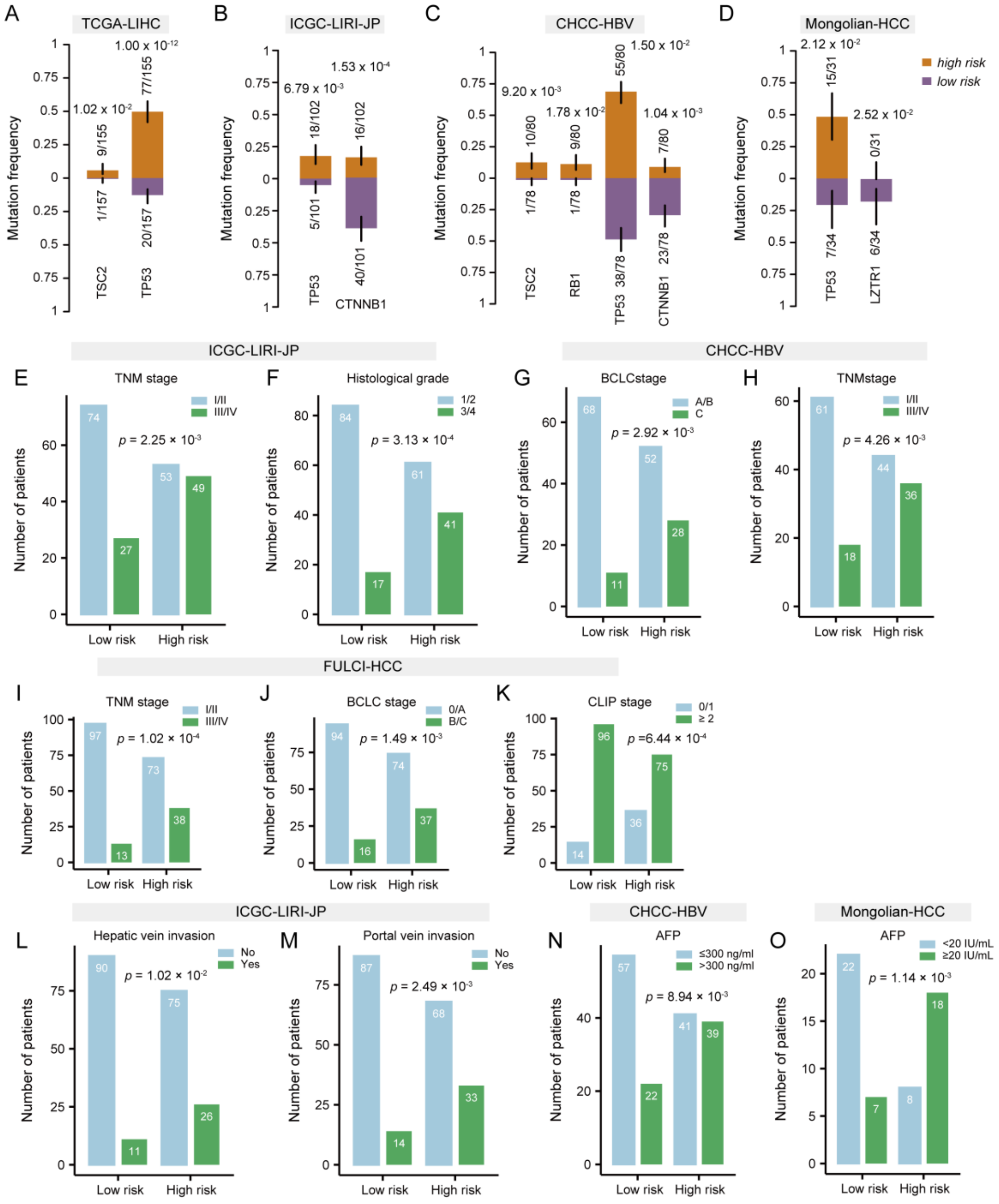

Figure S6. Validation of associations between clinicopathological and molecular features of HCC samples and HCCEvoSig risk scores. (A-D) Stacked bar plots illustrating genes with significantly different mutation frequencies between HCCEvoSig high- and low-risk groups in the TCGA-LIHC (A), ICGC-LIRI-JP (B), CHCC-HBV (C) and Mongolian-HCC (D) cohorts. (E-F) Bar plots showing the distribution of HCC patients with different TNM stages (E) and histological grades (F) in HCCEvoSig high- and low-risk groups within the ICGC-LIRI-JP cohort. (G-H) Bar plots depicting the distribution of HCC patients with different BCLC stages (G) and

TNM stages (H) in HCCEvoSig high- and low-risk groups within the CHCC-HBV cohort. (I-K) Bar plots displaying the distribution of HCC patients with different TNM stages (I), BCLC stages (J), and CLIP stages (K) in HCCEvoSig high- and low-risk groups within the FULCI-HCC cohort. (L-M) Bar plots showing the distribution of HCC patients with different hepatic vein invasion status (L) and portal vein invasion status (M) in HCCEvoSig high- and low-risk groups within the ICGC-LIRI-JP cohort. (N-O) Bar plots showing the distribution of HCC patients with different serum AFP levels in HCCEvoSig high- and low-risk groups within the CHCC-HBV (N) and FULCI-HCC (O) cohorts.

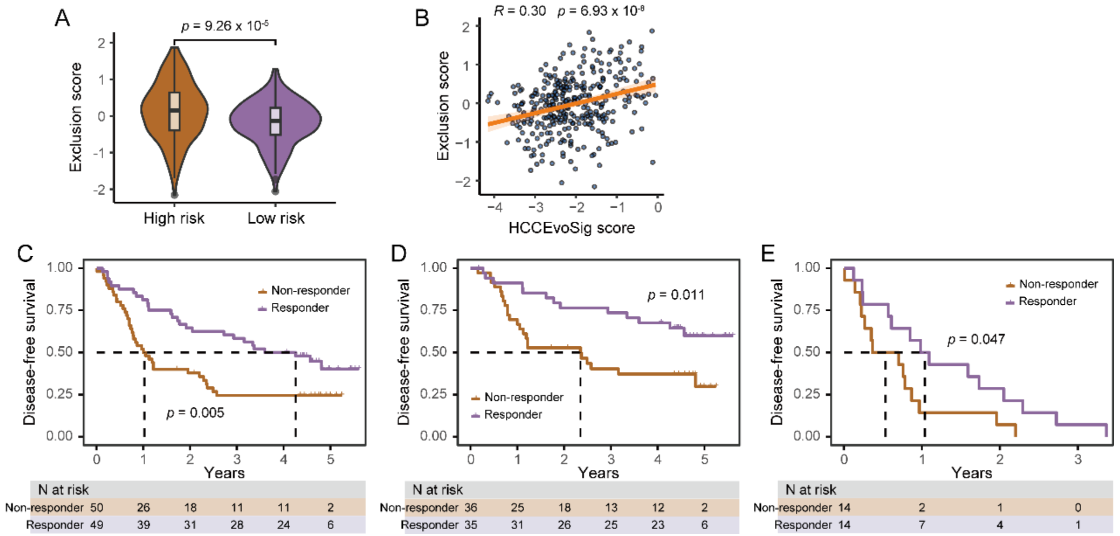

Figure S7. Predictive Performance of HCCEvoSig in Treatment Response. (A) Boxplot showing differences in exclusion scores between high and low HCCEvoSig risk groups. (B) Scatter plot illustrating the linear correlation between the HCCEvoSig risk score and exclusion score. (C-E) Comparison of disease-free survival between predicted responsive and non-responsive patients receiving either adjuvant or post-recurrence TACE (C), patients receiving adjuvant TACE (D), and patients receiving post-recurrence TACE (E). The dashed lines indicate the subgroup-specific median disease-free survival.

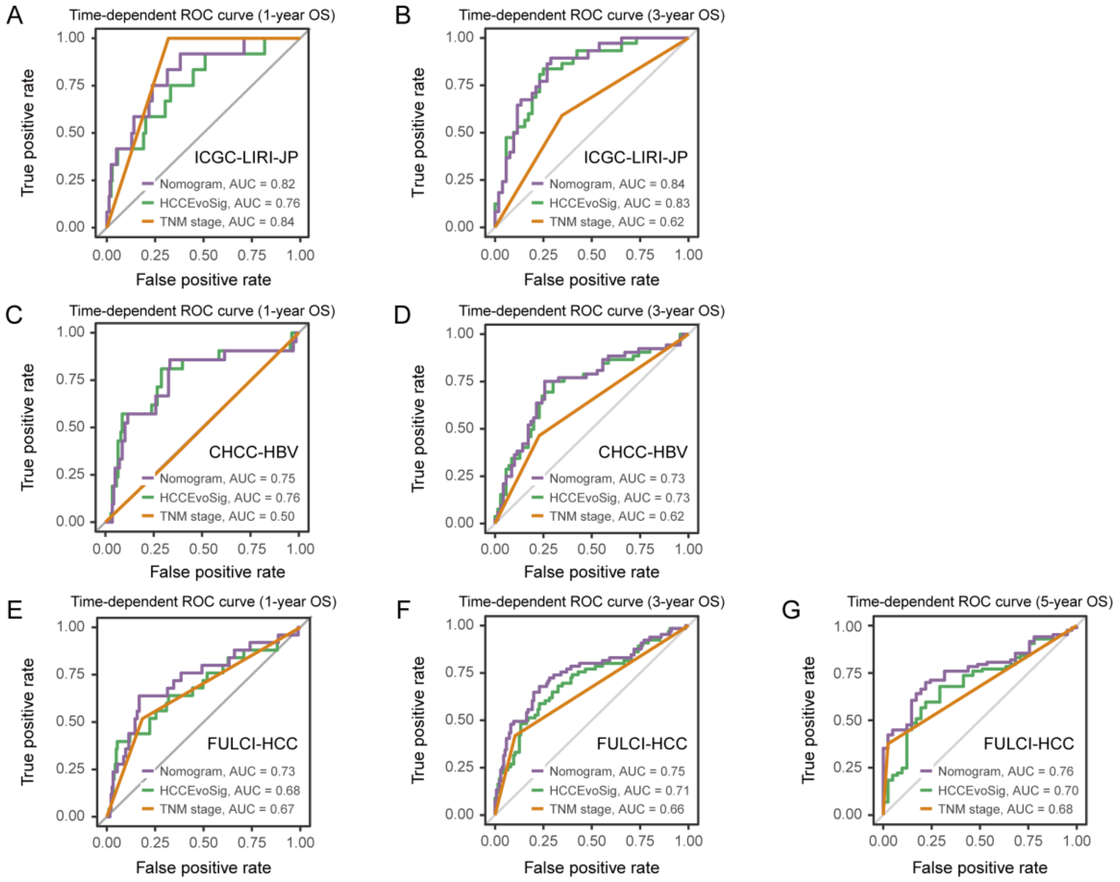

Figure S8. Performance of the nomogram for predicting 1-year, 3-year and 5-year survival of HCC patients. (A-G) Time-dependent ROC curves evaluating the sensitivity and specificity of the nomogram and individual independent predictive factors (HCCEvoSig and TNM stage) for predicting 1-year (A) and 3-year (B) survival in the independent RNA-seq-based ICGC-LIRI-JP cohort, 1-year (C) and 3-year (D) survival in the independent RNA-seq-based CHCC-HBV cohort, and 1-year (E), 3-year (F) and 5-year (G) survival in the independent microarray-based FULCI-HCC cohort. Different models are represented by different colored curves, with corresponding AUCs displayed in the lower right corner.

Supplementary tables

Table S1 Multiple-region expression datasets of HCC used in this study

Datasets	Number of patients	Number of samples	Mean (range) of samples per patient	Platform	Sources
MultiRRnaSeq1	7	33	4.7 (3-5)	Illumina	Losic et al., E-MTAB-5905
MultiRRnaSeq2	14	75	5.4 (3-10)	Illumina	Yang et al., https://zenodo.org/records/7336311
MultiRRnaSeq3	11	39	3.5 (3-5)	Illumina	Shen et al., GSE136711
MultiRArray	5	25	5 (5-5)	GPL6480	Shi et al., GSE92528

Table S2 The clinical follow-up datasets used in this study

Dataset	Platform	Data	WES-seq	Normal	Tumor	Paired	Sample	Patient	OS	RFS|PFS
TCGA-LIHC	Illumina	RNA-seq	Yes	42	323	42	367	323	323	323
ICGC-LIRI-JP	Illumina	RNA-seq	Yes	202	203	175	405	226	203	-
CHCC-HBV	Illumina	RNA-seq	Yes	159	159	159	318	159	159	159
Mongolian-HCC	Illumina	RNA-seq	Yes	70	70	70	140	70	70	-
FULCI-HCC	GPL3921	Affymetrix	No	220	225	214	445	231	221	221
NCI-HCC	GPL1528	NCI/ATC	No	0	140	0	140	140	112	-
Total	-	-	-	693	1120	660	1815	1149	1088	703

Table S3 Basic information on previously published prognostic gene expression signatures for HCC

PMID	Signature risk score formula	Gene size	Biological relevance of signature	Training cohort (Platform)	Validation cohort (Platform)
33251144	0.34900 × CNOT6 + 0.50277 × UPF3B + (-0.43143) × MRPL54 + (-0.21809) × ZC3H13 + (-0.46413) × IFIT5 + (-0.19919) × PPARGC1A	6	RNA Binding Protein-related	TCGA-LIHC (Illumina HiSeq 2000)	ICGC-LIRI-JP (Illumina HiSeq 2000)
32198063	0.253 × GINS2 + 0.286 × PTTG1 + 0.302 × ZWINT + 0.825 × CDC45 + 1.034 × MAD2L1 + 1.767 × BUB1B + 3.033 × KIF23 + (- 3.619) × PLK4 + (- 1.427) × OIP5 + (- 1.318) × KIF2 + (- 0.710) × CCCNB2 + (- 0.582) × DTL + (- 0.196) × BIRC5 + (- 0.186) × MCM4	14	Epithelial-related	TCGA-LIHC (Illumina HiSeq 2000)	FULCI-HCC (GPL3921); GSE76427 (GPL10558)
35123387	0.329 × BAK1 + 0.196 × BAX + 0.332 × CASP1 + (- 0.171) × CASP4 + (- 0.003) × CASP6 + 0.42 × GSDME + (- 0.392) × GZMA + (- 0.133) × GZMB + 0.179 × IL18 + (- 0.322) × TP53	10	Pyroptosis-related	TCGA-LIHC (Illumina HiSeq 2000)	GSE10186 (GPL5474)
34975331	0.400506 × DBF4 + 0.188240 × ARG2 + 0.204192 × SLC16A3	3	Microvascular invasion-related	TCGA-LIHC (Illumina HiSeq 2000)	ICGC-LIRI-JP (Illumina HiSeq 2000)
33828988	0.118 × SLC7A1 + 0.114 × RIPK2 + 0.113 × NOD2 + 0.022 × ADORA2B + 0.058 × MEP1A + 0.051 × ITGA5 + 0.016 × P2RX4 + 0.018 × SERPINE1	8	Inflammatory Response-related	TCGA-LIHC (Illumina HiSeq 2000)	ICGC-LIRI-JP (Illumina HiSeq 2000)
32903581	0.011 × ANLN + 0.014 × ENTPD2 + 0.001 × TRIP13 + 0.006 × PLAC8 + 0.001 × G6PD + (-2.037881e-06) × ADH1C	6	Energy Metabolism-related	TCGA-LIHC (Illumina HiSeq 2000)	GSE15654 (GPL8432); FULCI-HCC (GPL3921); GSE76427 (GPL10558)
33033585	0.5904 × ARHGAP5 + 0.1713 × ETV4 + (- 0.5468) × MAP2K1 + (- 0.2910) × BTG1 + 0.2505 × ACKR3 + 0.6266 × CDK4	6	Histone modification-related	TCGA-LIHC (Illumina HiSeq 2000)	No independent validation
34676211	(- 0.1137) × SLCO2A1 + (- 0.2461) × RPS6KA2 + 0.0503 × EPHB6 + 0.0441 × SLC2A5 + (-0.9111) × PDZD4 + 0.1804 × CST2 + 0.1193 × MARVELD1 + 0.0266 × MAGEA6 + 0.0645 × SEMA6A	9	Genomic instability of mutation-derived	TCGA-LIHC (Illumina HiSeq 2000)	ICGC-LIRI-JP (Illumina HiSeq 2000)
31335995	0.5202 × H2AFX + 0.5561 × SQSTM1 + (- 0.7905) × ITM2A + 0.5082 × PFKP + 0.4272 × TPD52L1 + 0.3985 × ACSL4 + 0.4861 × STRN3 + (- 0.4412) × CPEB3	8	Competitive endogenous RNA (ceRNA) regulatory network-related	TCGA-LIHC (Illumina HiSeq 2000)	FULCI-HCC (GPL3921); GSE76427 (GPL10558)

34900672	(-0.039384857) × EHMT2 + (-0.054911705) × HNRNPL + 0.119336909 × EIF2S1 + (-0.058903674) × PPARGC1A + 0.401757994 × RRP8 + 0.270963075 × FOXK1 + 0.160686777 × CAD + 0.101894034 × FOXK2 + (-0.089665498) × MYBBP1A	9	Starvation-Related mRNA Signature	TCGA-LIHC (Illumina HiSeq 2000)	ICGC-LIRI-JP (Illumina HiSeq 2000)
25666192	1.294 × ANGPT2 + 0.966 × DLL4 + 0.726 × NETO2 + 0.624 × NR4A1 + 0.557 × ESM1	5	Neoangiogenesis-related	GSE54236 (GPL6480)	(Real time PCR)
33089373	0.007898 × *NDRG1* + 0.032016 × *FABP6* + 0.04243 × *MAPT* + 0.000435 × *HSP90AA1* + 0.014474 × *CD320* + 0.014227 × *CACYBP* + 0.003685 × *BRD8* + 0.001297 × *OSGIN1* + 0.003575 × *NRAS* + 0.018457 × *ISG20L2* + 0.02678 × *PSMD14*	11	Immune-related gene signature	TCGA-LIHC (Illumina HiSeq 2000)	FULCI-HCC (GPL3921)
35311113	0.0071 × MAP2 + 0.1866 × DYNC1H1 + (−0.0197) × CPS1 + (−0.0312) × PTPRB + 0.1226 × MKI67	5	Mutation-gene signature	TCGA-LIHC (Illumina HiSeq 2000)	ICGC-LIRI-JP (Illumina HiSeq 2000)
22105560	(- 0.349) × ACSL5 + (- 0.401) × ADH1B + (- 0.258) × ADH6 + 0.226 × ALDOA + (- 0.297) ×APOC3 + (- 0.269) × AQP9 + 0.242 × ARPC2 + (- 0.645) × BPHL + (- 0.519) × C1orf115 + (- 0.353) × C4BPB + (- 0.347) × CDO1 + (- 0.195) × CHI3L1 + (- 0.389) × COBLL1 + (- 0.772) × CRAT + (- 0.439) × CRYL1 + 0.0338 × CTSC + 0.296 × CXCR4 + (- 0.255) × CYB5A + (- 0.38) × CYP27A1 + (- 0.613) × CYP2J2 + (- 0.198) × CYP4F12 + 0.305 × DDIT4 + (- 0.382) × EPHX2 + 0.228 × ETV5 + (- 0.325) × F10 + 0.788 × F3 + (- 0.312) × F5 + (- 0.421) × GJB1 + (- 0.521) × GPHN + 0.414 × JPT1 + (- 0.567) × HNF4A + 0.0252 × IGFBP3 + 0.34 × IQGAP1 + (- 0.539) × IQGAP2 + (- 0.632) × ITPR2 + (- 0.514) × KHK + 0.53 × LAMB1 + (- 0.138) × LECT2 + (- 0.312) × MST1 + (- 0.435) × MTSS1 + (- 0.381) × PAH + 0.521 × PFKFB3 + (- 0.386) × PKLR + 0.358 × PKM + (- 0.256) × PLG + 0.0948 × PLOD2 + 0.134 × PPT1 + 0.878 × RALA + (- 0.392) × RGN + 0.255 × RGS1 + 0.268 × RGS2 + (- 0.258) × RNASE4 + (- 0.391) × SERPINA10 + (- 0.228) × SERPINC1 + (- 0.352) × SERPINF2 + (- 0.269) × SFTPC + (- 0.476) × SLC22A7 + (- 0.38) × SLC2A2 + (- 0.337) × SLC30A1 + 0.184 × SLC38A1 + 0.356 × SPHK1 + (- 0.351) × SULT2A1 + (- 0.294) × TBX3 + 0.321 × TM4SF1 + 0.416 × TSPAN3	65	Derived from the NCI proliferation signature and the Seoul National University (SNU) recurrence signature	NCI-HCC (GPL1528)	GSE16757 (GPL6102); GSE9843 (GPL570); FULCI-HCC (GPL3921); INSERM-HCC (GPL96)
23800896	0.123 × LY6H + 0.078 × PCBP2 + 0.060 × THAP7 + 0.050 × SLC1A4 + 0.042 × ILMN_1851092 + 0.036 × TSPY3 + 0.033 × UBE2J2 + 0.023 × SLC16A10 + 0.023 × TMEM151A + 0.022 × FAM13A + 0.012 × HEY1 + 0.010 × RNF138 + 0.006 × MCM10 + 0.006 × ILMN_1868912 +	30	DFS gene signature	GSE36376 (GPL10558)	GSE25097 (GPL10687)

	0.005 × RGS12 + 0.004 × TDRD9 + (-0.003) × STBD1 + (-0.010) × CXCL10 + (-0.017) × TMEM187 + (-0.017) ×MAMDC4 + (-0.019) × MTFMT + (-0.019) × FAM126B + (-0.024) ×CTSH + (-0.025) × ID1 + (-0.026) × CD6 + (-0.035) × SLAMF9 + (-0.036) × MNDA + (-0.043) × SLCO2A1 + (-0.057) × LARGE1 + (-0.078) × TMEM187				

Table S4 Gene ontology enrichment analysis of EvoGenes

ID	Description	*p* Value	FDR
GO:0006805	xenobiotic metabolic process	6.43E-19	2.69E-15
GO:0071466	cellular response to xenobiotic stimulus	1.28E-18	2.69E-15
GO:0008202	steroid metabolic process	2.09E-16	2.93E-13
GO:0120254	olefinic compound metabolic process	6.73E-16	6.13E-13
GO:0009410	response to xenobiotic stimulus	7.30E-16	6.13E-13
GO:0046394	carboxylic acid biosynthetic process	1.41E-14	9.90E-12
GO:0016053	organic acid biosynthetic process	1.87E-14	1.12E-11
GO:0006631	fatty acid metabolic process	1.58E-13	8.27E-11
GO:0042445	hormone metabolic process	3.47E-13	1.62E-10
GO:0006935	chemotaxis	1.00E-12	4.22E-10
GO:0042330	taxis	1.15E-12	4.39E-10
GO:0042572	retinol metabolic process	7.74E-12	2.71E-09
GO:0009636	response to toxic substance	1.12E-11	3.62E-09
GO:1901605	alpha-amino acid metabolic process	1.41E-11	4.23E-09
GO:0034308	primary alcohol metabolic process	3.58E-11	1.00E-08
GO:0019373	epoxygenase P450 pathway	6.85E-11	1.80E-08
GO:0006721	terpenoid metabolic process	8.87E-11	2.16E-08
GO:0031667	response to nutrient levels	9.27E-11	2.16E-08
GO:0043434	response to peptide hormone	1.09E-10	2.40E-08
GO:0006066	alcohol metabolic process	1.87E-10	3.92E-08
GO:0060326	cell chemotaxis	5.46E-10	1.08E-07
GO:0001676	long-chain fatty acid metabolic process	5.81E-10	1.08E-07
GO:0006690	icosanoid metabolic process	5.94E-10	1.08E-07
GO:0098754	detoxification	6.89E-10	1.21E-07
GO:0019319	hexose biosynthetic process	3.28E-09	5.51E-07
GO:0044282	small molecule catabolic process	3.66E-09	5.91E-07
GO:0006520	amino acid metabolic process	4.23E-09	6.58E-07
GO:0070372	regulation of ERK1 and ERK2 cascade	4.54E-09	6.81E-07
GO:0050921	positive regulation of chemotaxis	6.78E-09	9.81E-07
GO:0001523	retinoid metabolic process	7.09E-09	9.93E-07
GO:0033559	unsaturated fatty acid metabolic process	8.97E-09	1.18E-06
GO:0030595	leukocyte chemotaxis	8.99E-09	1.18E-06
GO:0002687	positive regulation of leukocyte migration	1.05E-08	1.33E-06
GO:0016101	diterpenoid metabolic process	1.14E-08	1.37E-06
GO:0006720	isoprenoid metabolic process	1.14E-08	1.37E-06
GO:0046364	monosaccharide biosynthetic process	1.21E-08	1.41E-06
GO:0006094	gluconeogenesis	1.78E-08	1.98E-06
GO:0010043	response to zinc ion	1.79E-08	1.98E-06
GO:0070371	ERK1 and ERK2 cascade	2.05E-08	2.21E-06
GO:0062012	regulation of small molecule metabolic process	2.18E-08	2.26E-06
GO:0006869	lipid transport	2.20E-08	2.26E-06
GO:0010273	detoxification of copper ion	3.15E-08	3.08E-06

GO:1990169	stress response to copper ion	3.15E-08	3.08E-06
GO:0002688	regulation of leukocyte chemotaxis	3.39E-08	3.24E-06
GO:0002690	positive regulation of leukocyte chemotaxis	4.16E-08	3.88E-06
GO:0015711	organic anion transport	4.57E-08	4.17E-06
GO:0050900	leukocyte migration	4.73E-08	4.22E-06
GO:0097529	myeloid leukocyte migration	4.92E-08	4.30E-06
GO:1901607	alpha-amino acid biosynthetic process	5.20E-08	4.46E-06
GO:0006694	steroid biosynthetic process	5.42E-08	4.55E-06
GO:0006706	steroid catabolic process	5.73E-08	4.72E-06
GO:0072330	monocarboxylic acid biosynthetic process	6.82E-08	5.51E-06
GO:0051591	response to cAMP	7.11E-08	5.63E-06
GO:0006109	regulation of carbohydrate metabolic process	7.65E-08	5.95E-06
GO:0002685	regulation of leukocyte migration	9.22E-08	7.04E-06
GO:0016054	organic acid catabolic process	1.01E-07	7.43E-06
GO:0046395	carboxylic acid catabolic process	1.01E-07	7.43E-06
GO:0016125	sterol metabolic process	1.03E-07	7.47E-06
GO:0050920	regulation of chemotaxis	1.07E-07	7.61E-06
GO:0097305	response to alcohol	1.16E-07	8.12E-06
GO:0071276	cellular response to cadmium ion	1.19E-07	8.18E-06
GO:0030198	extracellular matrix organization	1.26E-07	8.56E-06
GO:0043062	extracellular structure organization	1.34E-07	8.94E-06
GO:0045229	external encapsulating structure organization	1.42E-07	9.34E-06
GO:0097006	regulation of plasma lipoprotein particle levels	1.63E-07	1.05E-05
GO:0015718	monocarboxylic acid transport	1.82E-07	1.14E-05
GO:0008652	amino acid biosynthetic process	1.84E-07	1.14E-05
GO:1901653	cellular response to peptide	1.85E-07	1.14E-05
GO:0090025	regulation of monocyte chemotaxis	2.10E-07	1.27E-05
GO:1901606	alpha-amino acid catabolic process	2.11E-07	1.27E-05
GO:0015850	organic hydroxy compound transport	2.24E-07	1.33E-05
GO:0009064	glutamine family amino acid metabolic process	2.45E-07	1.42E-05
GO:0045834	positive regulation of lipid metabolic process	2.46E-07	1.42E-05
GO:1903131	mononuclear cell differentiation	2.85E-07	1.62E-05
GO:0097501	stress response to metal ion	2.90E-07	1.62E-05
GO:0042180	cellular ketone metabolic process	3.10E-07	1.71E-05
GO:0009063	amino acid catabolic process	3.34E-07	1.82E-05
GO:0033762	response to glucagon	4.17E-07	2.22E-05
GO:0061687	detoxification of inorganic compound	4.17E-07	2.22E-05
GO:0002573	myeloid leukocyte differentiation	5.79E-07	3.04E-05
GO:0006959	humoral immune response	6.32E-07	3.28E-05
GO:0019369	arachidonic acid metabolic process	7.42E-07	3.80E-05
GO:0051346	negative regulation of hydrolase activity	7.73E-07	3.86E-05
GO:0070374	positive regulation of ERK1 and ERK2 cascade	7.73E-07	3.86E-05
GO:0071294	cellular response to zinc ion	8.13E-07	4.01E-05
GO:0071375	cellular response to peptide hormone stimulus	9.45E-07	4.61E-05

GO:0016042	lipid catabolic process	9.72E-07	4.69E-05
GO:0010038	response to metal ion	1.03E-06	4.90E-05
GO:0002526	acute inflammatory response	1.10E-06	5.20E-05
GO:0032868	response to insulin	1.25E-06	5.82E-05
GO:0071280	cellular response to copper ion	1.48E-06	6.85E-05
GO:0046688	response to copper ion	1.61E-06	7.33E-05
GO:0006633	fatty acid biosynthetic process	1.81E-06	8.17E-05
GO:0071621	granulocyte chemotaxis	1.86E-06	8.33E-05
GO:0030199	collagen fibril organization	2.20E-06	9.73E-05
GO:0050727	regulation of inflammatory response	2.22E-06	9.73E-05
GO:0046942	carboxylic acid transport	2.33E-06	1.01E-04
GO:0015849	organic acid transport	2.45E-06	1.05E-04
GO:0006006	glucose metabolic process	2.55E-06	1.08E-04
GO:0048545	response to steroid hormone	2.74E-06	1.15E-04

The 100 most significantly enriched GO terms were shown.

Table S5 KEGG enrichment analysis of EvoGenes

**ID**	**Description**	***p* Value**	**FDR**
hsa00830	Retinol metabolism	3.23E-11	6.20E-09
hsa00980	Metabolism of xenobiotics by cytochrome P450	4.53E-11	6.20E-09
hsa05204	Chemical carcinogenesis - DNA adducts	6.82E-11	6.23E-09
hsa00982	Drug metabolism - cytochrome P450	1.10E-10	7.52E-09
hsa04976	Bile secretion	2.56E-08	1.40E-06
hsa00140	Steroid hormone biosynthesis	8.53E-08	3.90E-06
hsa01230	Biosynthesis of amino acids	4.70E-06	1.84E-04
hsa03320	PPAR signaling pathway	5.42E-06	1.86E-04
hsa00350	Tyrosine metabolism	1.57E-05	4.77E-04
hsa04512	ECM-receptor interaction	2.84E-05	7.79E-04
hsa00220	Arginine biosynthesis	7.15E-05	1.78E-03
hsa04061	Viral protein interaction with cytokine and cytokine receptor	9.14E-05	2.09E-03
hsa04610	Complement and coagulation cascades	1.24E-04	2.61E-03
hsa00120	Primary bile acid biosynthesis	1.58E-04	3.10E-03
hsa00983	Drug metabolism - other enzymes	2.78E-04	5.08E-03
hsa04510	Focal adhesion	3.26E-04	5.59E-03
hsa04933	AGE-RAGE signaling pathway in diabetic complications	4.22E-04	6.81E-03
hsa00051	Fructose and mannose metabolism	7.05E-04	1.07E-02
hsa04978	Mineral absorption	7.53E-04	1.09E-02
hsa04979	Cholesterol metabolism	1.22E-03	1.67E-02
hsa00270	Cysteine and methionine metabolism	1.37E-03	1.75E-02
hsa00010	Glycolysis / Gluconeogenesis	1.41E-03	1.75E-02
hsa04668	TNF signaling pathway	1.67E-03	1.99E-02
hsa05417	Lipid and atherosclerosis	1.81E-03	2.04E-02
hsa05146	Amoebiasis	1.86E-03	2.04E-02

hsa00260	Glycine, serine and threonine metabolism	1.95E-03	2.05E-02
hsa04974	Protein digestion and absorption	2.15E-03	2.19E-02
hsa00480	Glutathione metabolism	2.89E-03	2.82E-02
hsa04151	PI3K-Akt signaling pathway	3.52E-03	3.33E-02
hsa04820	Cytoskeleton in muscle cells	3.71E-03	3.38E-02
hsa04060	Cytokine-cytokine receptor interaction	3.91E-03	3.38E-02
hsa00620	Pyruvate metabolism	3.95E-03	3.38E-02
hsa00590	Arachidonic acid metabolism	4.19E-03	3.43E-02
hsa00770	Pantothenate and CoA biosynthesis	4.25E-03	3.43E-02
hsa04670	Leukocyte transendothelial migration	4.46E-03	3.49E-02
hsa00040	Pentose and glucuronate interconversions	5.82E-03	4.43E-02
hsa00250	Alanine, aspartate and glutamate metabolism	6.56E-03	4.86E-02

Table S6 The detailed ingredients of HCCEvoSig

Gene ID	Gene symbol	Description	Risk coefficient	CancerLivER	RefMarker	Co-index
127	ADH4	alcohol dehydrogenase 4 (class II), pi polypeptide	0.01392683	YES	NO	4
991	CDC20	cell division cycle 20	0.13985825	NO	NO	20
10878	CFHR3	complement factor H related 3	-0.10684918	NO	NO	4
1559	CYP2C9	cytochrome P450 family 2 subfamily C member 9	-0.01570452	YES	NO	2
10268	RAMP3	receptor activity modifying protein 3	-0.22219705	NO	NO	3
8608	RDH16	retinol dehydrogenase 16	-0.04192664	NO	NO	2
5054	SERPINE1	serpin family E member 1	0.06911912	YES	YES	7
55002	SLC16A11	solute carrier family 16 member 11	-0.16745754	NO	NO	0
6696	SPP1	secreted phosphoprotein 1	0.07150320	YES	NO	20
6694	SPP2	secreted phosphoprotein 2	-0.01011339	NO	NO	1
388610	TRNP1	TMF1 regulated nuclear protein 1	0.02833318	NO	NO	0

CancerLivER represents if the targeted genes (ingredients of HCCEvoSig) are included in the biomarkers of the CancerLivER database that includes more than 594 liver cancer biomarkers.

RefMarker represents if the targeted genes (ingredients of HCCEvoSig) are included in the gene set composed of genes from 15 gene expression signatures of liver cancer that we collected from published articles.

Co-index represents the number of co-occurrences of the targeted gene (ingredient of HCCEvoSig, appearing in the title of article) and hepatocellular carcinoma (appearing in the title/abstract of article) in scientific literature in the PubMed database.

'YES' indicates presence, and 'NO' indicates absence.

Table S7 Differential expression of HCCEvoSig genes in epithelial cells, endothelial cells, fibroblasts and macrophages between tumor core and adjacent non-tumor tissue

	p_val	avg_log2FC	pct.1	pct.2	p_val_adj
**Epithelial cells**					
	**p_val**	**avg_log2FC**	**pct.1**	**pct.2**	**p_val_adj**
RDH16	2.87E-82	-1.1876	0.027	0.359	6.57E-78
SPP2	2.43E-33	-0.9033	0.117	0.435	5.57E-29
ADH4	1.03E-31	-1.1367	0.193	0.555	2.35E-27
CYP2C9	2.13E-23	-1.1671	0.214	0.498	4.86E-19
CFHR3	4.81E-19	-0.4569	0.024	0.144	1.10E-14
TRNP1	9.63E-06	0.2799	0.136	0.029	2.20E-01
CDC20	6.09E-05	0.5786	0.098	0.014	1.00E+00
SERPINE1	2.08E-04	-0.2124	0.087	0.167	1.00E+00
SPP1	1.33E-01	1.6023	0.225	0.196	1.00E+00
**Endothelial cells**					
	**p_val**	**avg_log2FC**	**pct.1**	**pct.2**	**p_val_adj**
RAMP3	1.64E-07	-0.5981	0.478	0.643	3.75E-03
**Fibroblasts**					
	**p_val**	**avg_log2FC**	**pct.1**	**pct.2**	**p_val_adj**
SERPINE1	4.97E-03	0.4577	0.332	0.159	1.00E+00
**Macrophages**					
	**p_val**	**avg_log2FC**	**pct.1**	**pct.2**	**p_val_adj**
SPP1	5.23E-191	4.3769	0.41	0.008	1.20E-186

Table S8 Differential expression of HCCEvoSig genes in epithelial cells, endothelial cells, fibroblasts and macrophages between tumor core and adjacent non-tumor tissue in HCC

	p_val	avg_log2FC	pct.1	pct.2	p_val_adj
**Epithelial cells**					
	**p_val**	**avg_log2FC**	**pct.1**	**pct.2**	**p_val_adj**
RDH16	2.00E-67	-1.3571	0.025	0.406	4.57E-63
ADH4	8.19E-25	-1.0834	0.234	0.65	1.87E-20
SPP2	2.18E-24	-0.9295	0.142	0.497	4.98E-20
CYP2C9	2.79E-18	-1.1376	0.255	0.566	6.39E-14
CFHR3	3.23E-10	-0.4849	0.027	0.133	7.40E-06
TRNP1	1.74E-05	0.2281	0.152	0.021	3.98E-01
SERPINE1	7.74E-05	-0.4204	0.101	0.21	1.00E+00
CDC20	3.30E-04	0.5410	0.122	0.021	1.00E+00
SPP1	3.34E-02	1.9311	0.243	0.182	1.00E+00
**Endothelial cells**					
	**p_val**	**avg_log2FC**	**pct.1**	**pct.2**	**p_val_adj**

RAMP3	9.39E-08	-0.8526	0.483	0.672	2.15E-03
		**Fibroblasts**			
	**p_val**	**avg_log2FC**	**pct.1**	**pct.2**	**p_val_adj**
SERPINE1	7.33E-01	-0.6380	0.163	0.132	1.00E+00
		**Macrophages**			
	**p_val**	**avg_log2FC**	**pct.1**	**pct.2**	**p_val_adj**
SPP1	2.16E-237	4.7684	0.508	0.005	4.94E-233

Table S9 Differential expression of HCCEvoSig genes between malignant and non-malignat epithelial cells

	**p_val**	**avg_log2FC**	**pct.1**	**pct.2**	**p_val_adj**
ADH4	1.86E-119	-2.2260	0.065	0.492	4.26E-115
SPP2	5.26E-93	-1.4144	0.027	0.364	1.20E-88
CYP2C9	6.30E-46	-1.4193	0.162	0.401	1.44E-41
RDH16	3.88E-40	-0.5314	0.008	0.16	8.87E-36
TRNP1	4.05E-35	0.2179	0.202	0.027	9.26E-31
SPP1	1.97E-31	2.1295	0.297	0.101	4.51E-27
CFHR3	2.37E-18	-0.1860	0.009	0.086	5.41E-14
CDC20	9.00E-15	0.3935	0.128	0.034	2.06E-10

Table S10 Differential expression of HCCEvoSig genes between malignant and non-malignat epithelial cells in HCC

	**p_val**	**avg_log2FC**	**pct.1**	**pct.2**	**p_val_adj**
ADH4	1.95E-88	-2.2174	0.098	0.57	4.47E-84
SPP2	2.75E-75	-1.5939	0.039	0.44	6.30E-71
CYP2C9	3.55E-43	-1.5941	0.177	0.473	8.12E-39
RDH16	3.27E-32	-0.6398	0.006	0.184	7.47E-28
TRNP1	4.91E-28	0.2475	0.218	0.022	1.12E-23
SPP1	2.14E-25	2.1880	0.337	0.111	4.91E-21
CDC20	9.78E-15	0.4938	0.162	0.034	2.24E-10
CFHR3	9.90E-12	-0.2055	0.013	0.091	2.27E-07

Table S11 Univariate and multivariate Cox regression analysis for overall survival of HCC in the TCGA-LIHC discovery cohort (n = 323)

Characteristics	Number of patients	Univariate mode		Multivariate mode	
		HR (95% CI)	P-value	HR (95% CI)	P-value
**Age**					
≥60 vs. <60	323	1.08 (0.75-1.57)	0.6764	1.40 (0.76-2.59)	0.2850
**Gender**					
Male vs. Female	323	0.81 (0.56-1.19)	0.2872	0.93 (0.47-1.83)	0.8327
**TNM stage**					
III/IV vs. I/II	306	**2.86 (1.95-4.21)**	**<0.0001**	**2.25 (1.19-4.26)**	**0.0124**
**Histological grade**					
3/4 vs. 1/2	320	1.06 (0.72-1.55)	0.7716	1.14 (0.62-2.10)	0.6818
**Cirrhosis**					
Yes vs. No	189	0.90 (0.51-1.61)	0.7254	1.37 (0.71-2.67)	0.3496
**AFP**					
≥300 vs. <300 ng/ml	248	1.09 (0.66-1.82)	0.7312	1.03 (0.51-2.07)	0.9442
**AUGUR risk**					
High vs. Low	323	**3.40 (2.27-5.09)**	**<0.0001**	**3.11 (1.60-6.06)**	**0.0008**

The bold values represent P-value less than 0.05. P-value < 0.05 is defined as statistical significance.

Table S12 Univariate and multivariate Cox regression analysis for overall survival of HCC in the ICGC-LIRI-JP validation cohort (n = 203)

Characteristics	Number of patients	Univariate mode		Multivariate mode	
		HR (95% CI)	P-value	HR (95% CI)	P-value
**Age**					
≥60 vs. <60	203	0.98 (0.43-2.24)	0.9532	0.82 (0.35-1.92)	0.6419
**Gender**					
Male vs. Female	203	0.53 (0.26-1.07)	0.0751	0.36 (0.16-0.79)	0.0111
**TNM stage**					
III/IV vs. I/II	203	**2.65 (1.36-5.19)**	**0.0044**	**2.85 (1.33-6.09)**	**0.0069**
**Histological grade**					
3/4 vs. 1/2	203	**2.86 (1.47-5.56)**	**0.0020**	1.78 (0.88-3.60)	0.1064
**Cirrhosis**					
Yes vs. No	202	1.24 (0.63-2.42)	0.5336	1.28 (0.65-2.53)	0.4789
**AUGUR risk**					
High vs. Low	203	**5.06 (2.20-11.65)**	**0.0001**	**3.71 (1.56-8.86)**	**0.0031**

The bold values represent P-value less than 0.05. P-value < 0.05 is defined as statistical significance.

Table S13 Univariate and multivariate Cox regression analysis for overall survival of HCC in the CHCC-HBV validation cohort (n = 159)

Characteristics	Number of patients	Univariate mode		Multivariate mode	
		HR (95% CI)	P-value	HR (95% CI)	P-value
**Age**					
≥60 vs. <60	159	0.67 (0.37-1.21)	0.1867	0.75 (0.40-1.39)	0.3556
**Gender**					
Male vs. Female	159	0.76 (0.41-1.41)	0.3806	1.01 (0.51-1.99)	0.9782
**Cirrhosis**					
Yes vs. No	159	1.28 (0.70-2.35)	0.4220	1.29 (0.69-2.40)	0.4201
**TNM stage**					
III/IV vs. I/II	159	**1.74 (1.02-2.95)**	**0.0408**	1.12 (0.63-2.00)	0.6922
**AFP**					
>300 vs. ≤300 ng/ml	159	**3.13 (1.83-5.34)**	**<0.0001**	**2.59 (1.47-4.57)**	**0.0010**
**AUGUR risk**					
High vs. Low	159	**4.20 (2.26-7.82)**	**<0.0001**	**3.45 (1.82-6.55)**	**0.0002**

The bold values represent P-value less than 0.05. P-value < 0.05 is defined as statistical significance.

Table S14 Univariate and multivariate Cox regression analysis for overall survival of HCC in the Mongolian-HCC validation cohort (n = 70)

Characteristics	Number of patients	Univariate mode		Multivariate mode	
		HR (95% CI)	P-value	HR (95% CI)	P-value
**Age**					
≥60 vs. <60	69	0.78 (0.34-1.77)	0.5494	1.5495 (0.517-4.6435)	0.4342
**Gender**					
Male vs. Female	69	1.90 (0.80-4.47)	0.1443	2.77 (0.94-8.16)	0.0646
**Cirrhosis**					
Yes vs. No	57	**4.63 (1.52-14.14)**	**0.0071**	**3.87 (0.96-15.65)**	**0.0579**
**TNM stage**					
III/IV vs. I/II	54	**5.03 (1.15-22.01)**	**0.0319**	**3.80 (0.82-17.62)**	**0.0885**
**AFP**					
Abnormal vs. Normal	55	2.07 (0.80-5.34)	0.1325	1.07 (0.28-4.04)	0.9180
**AUGUR risk**					
High vs. Low	70	**2.88 (1.18-7.02)**	**0.0197**	2.57 (0.63-10.50)	0.1875

The bold values represent P-value less than 0.05. P-value < 0.05 is defined as statistical significance.

Table S15 Univariate and multivariate Cox regression analysis for overall survival of HCC in the FULCI-HCC validation cohort (n = 221)

Characteristics	Number of patients	Univariate mode		Multivariate mode	
		HR (95% CI)	P-value	HR (95% CI)	P-value
**Age**					
≥60 vs. <60	221	0.86 (0.49-1.49)	0.5831	1.03 (0.59-1.82)	0.9120
**Gender**					
Male vs. Female	221	1.70 (0.82-3.52)	0.1533	1.40 (0.67-2.95)	0.3711
**Cirrhosis**					
Yes vs. No	221	**4.62 (1.14-18.80)**	**0.0324**	2.99 (0.72-12.39)	0.1313
**TNM stage**					
III/IV vs. I/II	221	**3.42 (2.19-5.35)**	**<0.0001**	**2.39 (1.47-3.88)**	**0.0004**
**AFP**					
>300 vs. ≤300 ng/ml	218	**1.63 (1.06-2.50)**	**0.0250**	1.20 (0.77-1.88)	0.4233
**AUGUR risk**					
High vs. Low	221	**2.84 (1.80-4.50)**	**<0.0001**	**2.05 (1.26-3.34)**	**0.0038**

The bold values represent P-value less than 0.05. P-value < 0.05 is defined as statistical significance.

Tables S16 Univariate and multivariate Cox regression analysis for overall survival of HCC in the CHCC-HBV validation cohort (n = 159)

Characteristics	Number of patients	Univariate mode		Multivariate mode	
		HR (95% CI)	P-value	HR (95% CI)	P-value
**Age**					
≥60 vs. <60	159	0.67 (0.37-1.21)	0.1867	0.79 (0.42-1.46)	0.4506
**Gender**					
Male vs. Female	159	0.76 (0.41-1.41)	0.3806	0.95 (0.49-1.86)	0.8919
**Cirrhosis**					
Yes vs. No	159	1.28 (0.70-2.35)	0.4220	1.36 (0.72-2.55)	0.3401
**BCLC stage**					
C vs. A/B	159	**2.22 (1.29-3.84)**	**0.0041**	1.44 (0.79-2.61)	0.2338
**AFP**					
>300 vs. ≤300 ng/ml	159	**3.13 (1.83-5.34)**	**<0.0001**	**2.42 (1.36-4.30)**	**0.0026**
**AUGUR risk**					
High vs. Low	159	**4.20 (2.26-7.82)**	**<0.0001**	**3.37 (1.78-6.38)**	**0.0002**

The bold values represent P-value less than 0.05. P-value < 0.05 is defined as statistical significance.

Table S17 Univariate and multivariate Cox regression analysis for overall survival of HCC in the FULCI-HCC validation cohort (n = 221)

Characteristics	Number of patients	Univariate mode		Multivariate mode	
		HR (95% CI)	P-value	HR (95% CI)	P-value
**Age**					
≥60 vs. <60	221	0.86 (0.49-1.49)	0.5831	1.15 (0.65-2.05)	0.6281
**Gender**					
Male vs. Female	221	1.70 (0.82-3.52)	0.1533	1.51 (0.72-3.15)	0.2710
**Cirrhosis**					
Yes vs. No	221	**4.62 (1.14-18.80)**	**0.0324**	3.07 (0.74-12.74)	0.1226
**BCLC stage**					
B/C vs. 0/A	221	**3.46 (2.22-5.38)**	**<0.0001**	**2.67 (1.65-4.31)**	**0.0001**
**AFP**					
>300 vs. ≤300 ng/ml	218	**1.63 (1.06-2.50)**	**0.0250**	1.15 (0.74-1.81)	0.5311
**AUGUR risk**					
High vs. Low	221	**2.84(1.80-4.50)**	**<0.0001**	**2.15 (1.33-3.48)**	**0.0019**

The bold values represent P-value less than 0.05. P-value < 0.05 is defined as statistical significance.

Table S18 Univariate and multivariate Cox regression analysis for overall survival of HCC in the FULCI-HCC validation cohort (n = 221)

Characteristics	Number of patients	Univariate mode		Multivariate mode	
		HR (95% CI)	P-value	HR (95% CI)	P-value
**Age**					
≥60 vs. <60	221	0.86 (0.49-1.49)	0.5831	1.07 (0.60-1.90)	0.8159
**Gender**					
Male vs. Female	221	1.70 (0.82-3.52)	0.1533	1.42 (0.68-2.98)	0.3551
**Cirrhosis**					
Yes vs. No	221	**4.62 (1.14-18.80)**	**0.0324**	2.88 (0.69-11.96)	0.1449
**CLIP stage**					
≥2 vs. 0/1	221	**3.14 (2.02-4.87)**	**<0.0001**	**0.39 (0.23-0.68)**	**0.0009**
**AFP**					
>300 vs. ≤300 ng/ml	218	**1.63 (1.06-2.50)**	**0.0250**	0.93 (0.55-1.56)	0.7821
**AUGUR risk**					
High vs. Low	221	**2.84(1.80-4.50)**	**<0.0001**	**2.29 (1.42-3.69)**	**0.0007**

The bold values represent P-value less than 0.05. P-value < 0.05 is defined as statistical significance.

Table S19 Comparison of HCCEvoSig with 15 reported prognostic signatures

Models	1-year AUC (95% CI)	3-year AUC (95% CI)	5-year AUC (95% CI)	C-index (95% CI)	P-value
HCCEvoSig	0.83(0.77-0.89)	0.75(0.68-0.83)	0.73(0.64-0.82)	0.74(0.70-0.79)	-
PMID33251144	0.76(0.68-0.84)	0.73(0.65-0.80)	0.72(0.63-0.81)	0.69(0.64-0.75)	7.21E-03
PMID32198063	0.69(0.62-0.76)	0.64(0.55-0.72)	0.63(0.52-0.74)	0.62(0.57-0.67)	1.85E-06
PMID35123387	0.72(0.64-0.80)	0.68(0.60-0.76)	0.66(0.56-0.76)	0.67(0.62-0.73)	3.66E-03
PMID31335995	0.71(0.62-0.79)	0.66(0.57-0.74)	0.68(0.59-0.78)	0.66(0.61-0.72)	5.79E-04
PMID33033585	0.69(0.61-0.78)	0.67(0.59-0.75)	0.65(0.55-0.75)	0.66(0.61-0.72)	1.43E-03
PMID33828988	0.69(0.61-0.77)	0.63(0.54-0.71)	0.69(0.58-0.79)	0.66(0.60-0.71)	2.25E-05
PMID34676211	0.75(0.67-0.83)	0.71(0.63-0.79)	0.66(0.56-0.76)	0.68(0.63-0.74)	8.87E-03
PMID32903581	0.72(0.63-0.80)	0.67(0.59-0.75)	0.64(0.54-0.75)	0.66(0.61-0.72)	2.25E-05
PMID34900672	0.70(0.61-0.80)	0.69(0.60-0.77)	0.70(0.61-0.80)	0.69(0.63-0.75)	2.72E-02
PMID33089373	0.72(0.64-0.80)	0.68(0.59-0.76)	0.64(0.53-0.74)	0.67(0.62-0.72)	3.09E-03
PMID35311113	0.76(0.68-0.83)	0.69(0.62-0.77)	0.62(0.51-0.73)	0.68(0.63-0.74)	4.92E-04
PMID34975331	0.69(0.62-0.77)	0.74(0.66-0.81)	0.69(0.60-0.79)	0.67(0.62-0.72)	1.24E-03
PMID25666192	0.60(0.52-0.68)	0.55(0.46-0.64)	0.51(0.40-0.63)	0.56(0.51-0.61)	3.10E-07
PMID22105560	0.74(0.67-0.81)	0.66(0.58-0.74)	0.67(0.56-0.77)	0.67(0.62-0.72)	3.57E-05
PMID23800896	0.74(0.65-0.82)	0.72(0.64-0.79)	0.65(0.55-0.76)	0.68(0.62-0.73)	4.34E-03

The P-value represents the significance of the C-index comparison between HCCEvoSig and other signatures.

Table S20 GSEA of differentially expressed genes between high- and low-risk groups

ID	Description	NES	Pvalue	P.adjust	Qvalue
hsa00830	Retinol metabolism	-3.31	1.00E-10	2.63E-09	1.64E-09
hsa05204	Chemical carcinogenesis - DNA adducts	-3.30	1.00E-10	2.63E-09	1.64E-09
hsa00980	Metabolism of xenobiotics by cytochrome P450	-3.28	1.00E-10	2.63E-09	1.64E-09
hsa00982	Drug metabolism - cytochrome P450	-3.26	1.00E-10	2.63E-09	1.64E-09
hsa04610	Complement and coagulation cascades	-3.20	1.00E-10	2.63E-09	1.64E-09
hsa00071	Fatty acid degradation	-3.12	1.00E-10	2.63E-09	1.64E-09
hsa00140	Steroid hormone biosynthesis	-2.99	1.00E-10	2.63E-09	1.64E-09
hsa00380	Tryptophan metabolism	-2.99	1.00E-10	2.63E-09	1.64E-09
hsa00280	Valine, leucine and isoleucine degradation	-2.81	1.00E-10	2.63E-09	1.64E-09
hsa04146	Peroxisome	-2.80	1.00E-10	2.63E-09	1.64E-09
hsa03320	PPAR signaling pathway	-2.63	1.00E-10	2.63E-09	1.64E-09
hsa04976	Bile secretion	-2.53	1.00E-10	2.63E-09	1.64E-09
hsa04110	Cell cycle	1.96	1.00E-10	2.63E-09	1.64E-09
hsa00350	Tyrosine metabolism	-2.79	1.27E-10	3.10E-09	1.94E-09
hsa00260	Glycine, serine and threonine metabolism	-2.72	3.71E-10	8.28E-09	5.17E-09
hsa03040	Spliceosome	1.82	3.87E-10	8.28E-09	5.17E-09
hsa00591	Linoleic acid metabolism	-2.71	2.12E-09	4.26E-08	2.66E-08
hsa00650	Butanoate metabolism	-2.66	9.18E-09	1.74E-07	1.09E-07
hsa00120	Primary bile acid biosynthesis	-2.60	1.27E-08	2.29E-07	1.43E-07
hsa00983	Drug metabolism - other enzymes	-2.32	2.06E-08	3.52E-07	2.20E-07

hsa00620	Pyruvate metabolism	-2.52	2.33E-08	3.62E-07	2.26E-07
hsa01240	Biosynthesis of cofactors	-1.90	2.32E-08	3.62E-07	2.26E-07
hsa00220	Arginine biosynthesis	-2.50	1.19E-07	1.77E-06	1.11E-06
hsa00410	beta-Alanine metabolism	-2.50	3.17E-07	4.52E-06	2.82E-06
hsa03030	DNA replication	1.91	4.24E-07	5.80E-06	3.62E-06
hsa03010	Ribosome	1.65	9.33E-07	1.23E-05	7.67E-06
hsa04144	Endocytosis	1.53	1.01E-06	1.27E-05	7.96E-06
hsa00340	Histidine metabolism	-2.39	1.24E-06	1.51E-05	9.43E-06
hsa00630	Glyoxylate and dicarboxylate metabolism	-2.33	1.50E-06	1.77E-05	1.11E-05
hsa03082	ATP-dependent chromatin remodeling	1.65	1.72E-06	1.90E-05	1.19E-05
hsa05206	MicroRNAs in cancer	1.60	1.69E-06	1.90E-05	1.19E-05
hsa01200	Carbon metabolism	-1.93	2.05E-06	2.19E-05	1.37E-05
hsa00640	Propanoate metabolism	-2.35	2.56E-06	2.66E-05	1.66E-05
hsa00590	Arachidonic acid metabolism	-2.09	8.12E-06	8.17E-05	5.10E-05
hsa03013	Nucleocytoplasmic transport	1.64	9.31E-06	9.09E-05	5.68E-05
hsa05166	Human T-cell leukemia virus 1 infection	1.50	1.07E-05	1.02E-04	6.36E-05
hsa01210	2-Oxocarboxylic acid metabolism	-2.25	1.11E-05	1.02E-04	6.39E-05
hsa00053	Ascorbate and aldarate metabolism	-2.25	1.19E-05	1.05E-04	6.54E-05
hsa04979	Cholesterol metabolism	-2.13	1.19E-05	1.05E-04	6.54E-05
hsa04814	Motor proteins	1.52	1.93E-05	1.61E-04	1.00E-04
hsa05165	Human papillomavirus infection	1.40	1.91E-05	1.61E-04	1.00E-04
hsa00010	Glycolysis / Gluconeogenesis	-2.01	2.42E-05	1.97E-04	1.23E-04
hsa00670	One carbon pool by folate	-2.10	3.58E-05	2.85E-04	1.78E-04
hsa04218	Cellular senescence	1.51	3.72E-05	2.89E-04	1.81E-04
hsa04936	Alcoholic liver disease	-1.67	6.18E-05	4.70E-04	2.93E-04
hsa00250	Alanine, aspartate and glutamate metabolism	-2.02	6.77E-05	4.98E-04	3.11E-04
hsa03008	Ribosome biogenesis in eukaryotes	1.62	6.85E-05	4.98E-04	3.11E-04
hsa04975	Fat digestion and absorption	-2.07	7.52E-05	5.36E-04	3.35E-04
hsa01212	Fatty acid metabolism	-1.91	7.88E-05	5.50E-04	3.43E-04
hsa03015	mRNA surveillance pathway	1.56	8.58E-05	5.87E-04	3.67E-04
hsa03410	Base excision repair	1.64	1.12E-04	7.49E-04	4.68E-04
hsa05219	Bladder cancer	1.65	1.24E-04	8.02E-04	5.01E-04
hsa05203	Viral carcinogenesis	1.43	1.22E-04	8.02E-04	5.01E-04
hsa05132	Salmonella infection	1.42	1.33E-04	8.45E-04	5.28E-04
hsa04114	Oocyte meiosis	1.52	1.37E-04	8.54E-04	5.34E-04
hsa05100	Bacterial invasion of epithelial cells	1.59	1.57E-04	9.58E-04	5.98E-04
hsa00330	Arginine and proline metabolism	-1.93	2.06E-04	1.22E-03	7.64E-04
hsa04390	Hippo signaling pathway	1.46	2.07E-04	1.22E-03	7.64E-04
hsa05131	Shigellosis	1.42	2.15E-04	1.25E-03	7.80E-04
hsa05130	Pathogenic Escherichia coli infection	1.43	2.28E-04	1.30E-03	8.11E-04
hsa00270	Cysteine and methionine metabolism	-1.88	2.65E-04	1.48E-03	9.27E-04
hsa00020	Citrate cycle (TCA cycle)	-1.94	2.79E-04	1.54E-03	9.60E-04
hsa00040	Pentose and glucuronate interconversions	-1.96	2.97E-04	1.61E-03	1.01E-03
hsa04666	Fc gamma R-mediated phagocytosis	1.52	3.09E-04	1.65E-03	1.03E-03

hsa00860	Porphyrin metabolism	-1.88	3.79E-04	2.00E-03	1.25E-03
hsa01232	Nucleotide metabolism	1.49	5.24E-04	2.72E-03	1.70E-03
hsa03440	Homologous recombination	1.58	6.71E-04	3.42E-03	2.14E-03
hsa01230	Biosynthesis of amino acids	-1.71	7.37E-04	3.71E-03	2.32E-03
hsa03460	Fanconi anemia pathway	1.57	7.95E-04	3.94E-03	2.46E-03
hsa03083	Polycomb repressive complex	1.47	8.61E-04	4.21E-03	2.63E-03
hsa04115	p53 signaling pathway	1.51	9.78E-04	4.71E-03	2.94E-03
hsa04913	Ovarian steroidogenesis	-1.72	1.34E-03	6.35E-03	3.97E-03
hsa04120	Ubiquitin mediated proteolysis	1.42	1.47E-03	6.87E-03	4.29E-03
hsa04657	IL-17 signaling pathway	1.47	1.55E-03	7.18E-03	4.49E-03
hsa04914	Progesterone-mediated oocyte maturation	1.45	1.59E-03	7.25E-03	4.53E-03
hsa03018	RNA degradation	1.50	1.64E-03	7.38E-03	4.61E-03
hsa00360	Phenylalanine metabolism	-1.86	2.43E-03	1.08E-02	6.75E-03
hsa03420	Nucleotide excision repair	1.52	2.49E-03	1.09E-02	6.83E-03
hsa05211	Renal cell carcinoma	1.48	2.57E-03	1.11E-02	6.94E-03
hsa04142	Lysosome	1.41	2.73E-03	1.17E-02	7.30E-03
hsa04145	Phagosome	1.38	2.79E-03	1.18E-02	7.35E-03
hsa00970	Aminoacyl-tRNA biosynthesis	1.49	2.89E-03	1.20E-02	7.52E-03
hsa04920	Adipocytokine signaling pathway	-1.60	3.61E-03	1.49E-02	9.30E-03
hsa04530	Tight junction	1.36	3.72E-03	1.52E-02	9.47E-03
hsa04820	Cytoskeleton in muscle cells	1.30	3.85E-03	1.55E-02	9.69E-03
hsa04933	AGE-RAGE signaling pathway in diabetic complications	1.39	4.11E-03	1.63E-02	1.02E-02
hsa00785	Lipoic acid metabolism	-1.84	4.83E-03	1.86E-02	1.16E-02
hsa03430	Mismatch repair	1.58	4.85E-03	1.86E-02	1.16E-02
hsa05152	Tuberculosis	1.35	4.77E-03	1.86E-02	1.16E-02
hsa04922	Glucagon signaling pathway	-1.44	4.98E-03	1.87E-02	1.17E-02
hsa05135	Yersinia infection	1.38	4.95E-03	1.87E-02	1.17E-02
hsa05140	Leishmaniasis	1.42	5.60E-03	2.08E-02	1.30E-02
hsa04668	TNF signaling pathway	1.38	5.88E-03	2.16E-02	1.35E-02
hsa00430	Taurine and hypotaurine metabolism	-1.75	6.28E-03	2.25E-02	1.41E-02
hsa04981	Folate transport and metabolism	-1.69	6.30E-03	2.25E-02	1.41E-02
hsa04810	Regulation of actin cytoskeleton	1.28	6.32E-03	2.25E-02	1.41E-02
hsa04931	Insulin resistance	-1.42	6.55E-03	2.31E-02	1.44E-02
hsa04380	Osteoclast differentiation	1.35	7.60E-03	2.65E-02	1.66E-02
hsa05161	Hepatitis B	1.34	7.78E-03	2.69E-02	1.68E-02
hsa03020	RNA polymerase	1.48	9.24E-03	3.16E-02	1.98E-02
hsa05323	Rheumatoid arthritis	1.37	1.05E-02	3.55E-02	2.22E-02
hsa05170	Human immunodeficiency virus 1 infection	1.28	1.08E-02	3.62E-02	2.26E-02
hsa05230	Central carbon metabolism in cancer	1.41	1.10E-02	3.67E-02	2.29E-02
hsa04512	ECM-receptor interaction	1.35	1.25E-02	4.08E-02	2.55E-02
hsa04015	Rap1 signaling pathway	1.27	1.24E-02	4.08E-02	2.55E-02
hsa04727	GABAergic synapse	-1.40	1.27E-02	4.09E-02	2.55E-02
hsa05169	Epstein-Barr virus infection	1.29	1.29E-02	4.12E-02	2.58E-02

hsa04141	Protein processing in endoplasmic reticulum	1.31	1.35E-02	4.28E-02	2.68E-02
hsa05163	Human cytomegalovirus infection	1.27	1.45E-02	4.56E-02	2.85E-02
hsa04726	Serotonergic synapse	-1.35	1.52E-02	4.72E-02	2.95E-02

Table S21 The hallmark score of high- and low-risk HCCEvoSig group

Names	Mean score of the low-risk group	Mean score of the high-risk group	Pvalue	FDR
HALLMARK_G2M_CHECKPOINT	0.2864	0.3642	3.56E-25	1.78E-23
HALLMARK_MYC_TARGETS_V1	0.6834	0.7087	6.26E-23	1.57E-21
HALLMARK_E2F_TARGETS	0.3540	0.4320	2.19E-22	3.64E-21
HALLMARK_BILE_ACID_METABOLISM	0.4820	0.4173	5.08E-19	6.35E-18
HALLMARK_MITOTIC_SPINDLE	0.3065	0.3440	9.65E-19	9.65E-18
HALLMARK_FATTY_ACID_METABOLISM	0.5988	0.5624	9.95E-18	8.29E-17
HALLMARK_XENOBIOTIC_METABOLISM	0.6086	0.5646	8.22E-15	5.87E-14
HALLMARK_SPERMATOGENESIS	-0.1105	-0.0843	2.23E-14	1.39E-13
HALLMARK_ADIPOGENESIS	0.5771	0.5577	4.02E-14	2.23E-13
HALLMARK_PI3K_AKT_MTOR_SIGNALING	0.4229	0.4361	3.21E-12	1.61E-11
HALLMARK_DNA_REPAIR	0.5189	0.5342	5.06E-12	2.30E-11
HALLMARK_OXIDATIVE_PHOSPHORYLATION	0.7461	0.7343	8.64E-12	3.60E-11
HALLMARK_MTORC1_SIGNALING	0.5551	0.5739	3.15E-11	1.21E-10
HALLMARK_PEROXISOME	0.5243	0.5032	1.34E-10	4.79E-10
HALLMARK_GLYCOLYSIS	0.4339	0.4515	3.93E-10	1.31E-09
HALLMARK_UNFOLDED_PROTEIN_RESPONSE	0.5401	0.5518	1.23E-08	3.84E-08
HALLMARK_COAGULATION	0.5439	0.5204	2.36E-08	6.93E-08
HALLMARK_MYC_TARGETS_V2	0.4918	0.5167	7.51E-08	2.09E-07
HALLMARK_ESTROGEN_RESPONSE_LATE	0.2848	0.2969	2.20E-05	5.78E-05
HALLMARK_HEME_METABOLISM	0.3182	0.3079	4.95E-05	1.24E-04
HALLMARK_PANCREAS_BETA_CELLS	0.0209	0.0058	2.14E-04	5.10E-04
HALLMARK_UV_RESPONSE_UP	0.4672	0.4759	4.67E-04	1.06E-03
HALLMARK_P53_PATHWAY	0.4192	0.4289	8.07E-04	1.75E-03
HALLMARK_KRAS_SIGNALING_DN	-0.1382	-0.1450	1.18E-03	2.45E-03
HALLMARK_REACTIVE_OXYGEN_SPECIES_PATHWAY	0.5985	0.6072	2.98E-03	5.95E-03
HALLMARK_ANDROGEN_RESPONSE	0.4496	0.4399	3.51E-03	6.55E-03
HALLMARK_UV_RESPONSE_DN	0.2743	0.2603	3.54E-03	6.55E-03
HALLMARK_MYOGENESIS	0.1913	0.1807	1.29E-02	2.30E-02
HALLMARK_PROTEIN_SECRETION	0.5046	0.5124	1.67E-02	2.88E-02

Table S22 Comparison of the integrated nomogram model with any single independent predictive factor: HCCEvoSig and TNM stage

Dataset	Models	1-year AUC (95% CI)	3-year AUC (95% CI)	5-year AUC (95% CI)	C-index (95% CI)	P-value
TCGA-LIHC	Nomogram	0.83(0.77-0.90)	0.79(0.72-0.86)	0.78(0.69-0.87)	0.76 (0.71-0.81)	-
	HCCEvoSig	0.82(0.76-0.88)	0.75(0.68-0.82)	0.75(0.66-0.84)	0.74 (0.69-0.79)	1.02E-01
	TNM stage	0.69(0.61-0.76)	0.66(0.59-0.73)	0.63(0.55-0.71)	0.62 (0.57-0.67)	3.42E-14
ICGC-LIRI-JP	Nomogram	0.82(0.70-0.93)	0.84(0.76-0.93)	-	0.79 (0.72-0.86)	-
	HCCEvoSig	0.76(0.62-0.90)	0.83(0.74-0.92)	-	0.77 (0.69-0.85)	2.31E-02
	TNM stage	0.84(0.81-0.88)	0.62(0.51-0.73)	-	0.67(0.59-0.75)	8.76E-04
CHCC-HBV	Nomogram	0.75(0.63-0.88)	0.73(0.64-0.83)	-	0.72 (0.65-0.79)	-
	HCCEvoSig	0.76(0.64-0.89)	0.73(0.63-0.82)	-	0.72 (0.65-0.79)	9.65E-01
	TNM stage	0.50(0.39-0.60)	0.62(0.53-0.70)	-	0.56 (0.49-0.62)	4.29E-08
FULCI-HCC	Nomogram	0.73(0.61-0.84)	0.75(0.67-0.83)	0.76(0.68-0.84)	0.70 (0.64-0.76)	-
	HCCEvoSig	0.68(0.56-0.81)	0.71(0.63-0.79)	0.70(0.60-0.79)	0.66 (0.60-0.73)	1.31E-01
	TNM stage	0.67(0.56-0.77)	0.66(0.59-0.72)	0.68(0.62-0.73)	0.62 (0.57-0.67)	6.71E-03

P-value represents the significance of the C-index comparison between integrated nomogram with AUGUR or TNM stage.
